# NKCC1 controls GABAergic signaling and neuroblast migration in the postnatal forebrain

**DOI:** 10.1186/1749-8104-6-4

**Published:** 2011-02-01

**Authors:** Sheyla Mejia-Gervacio, Kerren Murray, Pierre-Marie Lledo

**Affiliations:** 1 Institut Pasteur, Laboratory for Perception and Memory, 25 rue du Dr. Roux, F-75724 Paris Cedex 15, France; 2 Centre National de la Recherche Scientifique (CNRS) Unité de Recherche Associée (URA) 2182, 75724 Paris, France

## Abstract

From an early postnatal period and throughout life there is a continuous production of olfactory bulb (OB) interneurons originating from neuronal precursors in the subventricular zone. To reach the OB circuits, immature neuroblasts migrate along the rostral migratory stream (RMS). In the present study, we employed cultured postnatal mouse forebrain slices and used lentiviral vectors to label neuronal precursors with GFP and to manipulate the expression levels of the Na-K-2Cl cotransporter NKCC1. We investigated the role of this Cl^- ^transporter in different stages of postnatal neurogenesis, including neuroblast migration and integration in the OB networks once they have reached the granule cell layer (GCL). We report that NKCC1 activity is necessary for maintaining normal migratory speed. Both pharmacological and genetic manipulations revealed that NKCC1 maintains high [Cl^-^]_i _and regulates the resting membrane potential of migratory neuroblasts whilst its functional expression is strongly reduced at the time cells reach the GCL. As in other developing systems, NKCC1 shapes GABA_A_-dependent signaling in the RMS neuroblasts. Also, we show that NKCC1 controls the migration of neuroblasts in the RMS. The present study indeed indicates that the latter effect results from a novel action of NKCC1 on the resting membrane potential, which is independent of GABA_A_-dependent signaling. All in all, our findings show that early stages of the postnatal recruitment of OB interneurons rely on precise, orchestrated mechanisms that depend on multiple actions of NKCC1.

## Background

The subventricular zone (SVZ) and the rostral migratory stream (RMS) contain neural progenitors that proliferate and migrate to the olfactory bulb (OB) throughout life [1]. In the OB, the precursors differentiate into two classes of interneurons: granule cells (GCs) and periglomerular cells (PGCs) [2-4], which populate deep and superficial layers of the OB, respectively.

The migration of neuroblasts in the RMS is a highly regulated process, and may determine the rate of integration of newborn neurons in the OB. A series of chemical factors have been identified as regulators of neuroblast migration in the RMS, including adhesion molecules [5], extracellular matrix molecules [6], and molecules with repulsive/attractive functions [7-9]. Moreover, migration in the SVZ-OB system is sensitive to the action of neurotransmitters [10,11], providing a basis for neural activity-dependent regulation of postnatal neurogenesis. In this regard, a previous study showed that neuroblast migration in the RMS is reduced by the tonic depolarizing action of gamma-aminobutyric acid (GABA) acting on GABA_A _receptors [12,13].

GABA_A _receptor activity affects the cell membrane potential, producing depolarizing or hyperpolarizing influences depending on the Cl^- ^gradient across the cell membrane, principally determined by the intracellular concentration of Cl^- ^([Cl^-^]_i_) [14-16]. [Cl^-^]_i _is tightly regulated by the activity of ionic transporters such as Na^+^-K^+^-2Cl^- ^(NKCC1) or K^+^-Cl^- ^(KCC2) [17-19], which, following the driving force of Na^+ ^and K^+^, have the tendency to increase or decrease, respectively, the [Cl^-^]_i _at rest.

NKCC1 is frequently expressed in developing central nervous system regions and its activity has been associated with depolarizing actions of GABA [19-23]. It is presently unknown if NKCC1 regulates the [Cl^-^]_i _in the RMS. This is an important issue since, in several regions, depolarizing GABA signaling appears early in development and can modulate neuronal excitability to promote neuronal maturation [21,24-27].

In the present study, we investigated whether NKCC1 shapes the action of GABA on the maturation of OB neuronal precursors and its influence on cellular motility. Our results indicate that the activation of GABA_A _receptors induces a strong positive driving force in migrating neuroblasts, which diminishes upon arrival of the cells at the GC layer (GCL). We identified NKCC1 as the uptake mechanism maintaining elevated [Cl^-^]_i _in migrating neuroblasts, thus shaping the action of GABA_A _receptors. Moreover, we report that the activity of NKCC1 is necessary to maintain normal migration speed and this process occurs independently of GABA_A_-signaling since NKCC1 controls the resting state of excitability in RMS neuroblasts.

## Results

### Forebrain organotypic slice cultures

**Figure 1:**
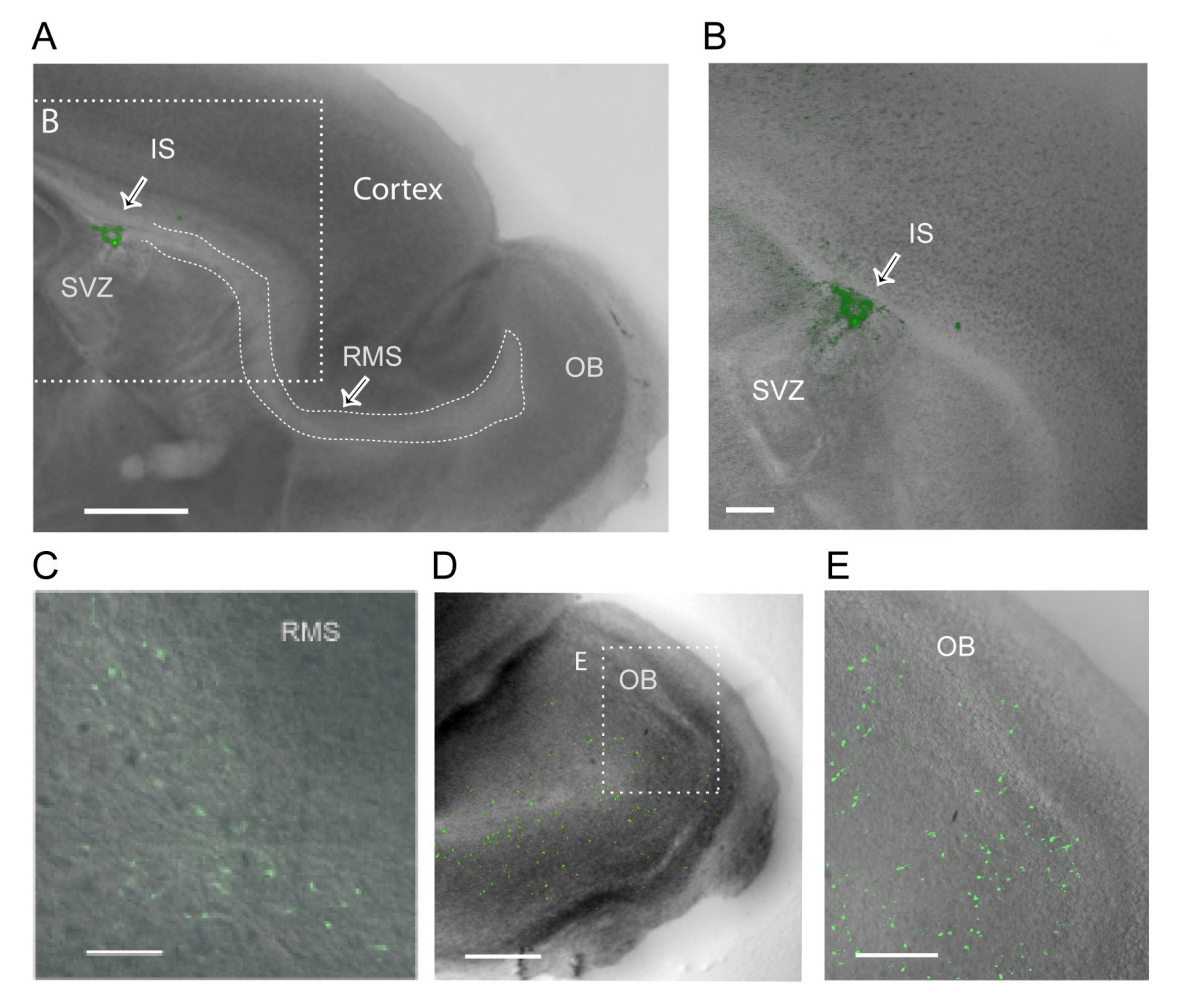
**The injection of lentiviral vectors in the SVZ of organotypic slice cultures gives rise to GFP**^**+ **^**migrating neuroblasts in the RMS and labeled neurons in all OB layers**. **(A) **Photomicrograph of a cultured mouse forebrain sagittal section containing the SVZ-RMS pathway, showing the viral injection site (IS) in the SVZ at 2 days post-injection (dpi). Scale bar: 800 μm. Inset. Approximate location of the image shown in (B). **(B) **Detail of the IS displayed in (A) at higher magnification. Scale bar: 200 μm. **(C) **Photomicrograph showing GFP^+ ^cells migrating in the RMS at 5 dpi. Scale bar: 100 μm. **(D) **Photomicrograph of the entire OB showing the arrival of GFP^+ ^cells at 5 dpi. Scale bar: 500 μm. The outline represents the approximate location of the magnification in (E). **(E) **Photomicrograph of the area outlined in (D), magnified to show GFP^+ ^cells scattered throughout all OB layers. The same slice culture used in (D) is shown at 11 dpi. Scale bar: 200 μm.

We developed an organotypic slice culture model that maintains the anatomical organization of the SVZ-OB system (Figure 1A) to study the migration and the first steps of integration of neuronal precursors in the OB circuit at early postnatal periods. For this study, the slices were obtained from 7-day-old mice and maintained for up to 12 days *in vitro *(div). Immunohistochemical staining performed at 6 div with antibodies directed against a neuronal precursor marker (doublecortin (DCX); n = 3 slices; Additional file 1) and more mature neuronal markers (NeuN, tyrosine hydroxylase (TH) and GABA; n = 3 slices for each; Additional files 1 and 2) were in agreement with the expected distribution patterns observed at similar postnatal ages, as visually compared to the Allen Institute for Brain Science atlas for P14 animals (and P7 when available) for TH and DCX. Similarly, our labeling corresponded to previously published data from adult slices for GABA in the RMS [12] and NeuN in the OB [28,29]. Together, these observations suggested that overall OB organization was preserved in our slice model.

A total of 132 cultured slices were transduced at 1 div with lentiviral vectors to induce the expression of GFP and to manipulate their levels of NKCC1 expression in SVZ precursor cells. After 1 to 2 days post-injection (dpi), strong GFP labeling was visible at the injection site (Figure 1A,B). Several GFP^+ ^cells were distributed along the RMS shortly after vector injection (>3 dpi; Figure 1C). These labeled cells displayed typical morphology of migrating neuroblasts, with a small and elongated cell body (6 to 8 μm), including leading and trailing processes [30]. The first GFP^+ ^cells reached the GCL 5 days after vector injection (Figure 1D). Six days after injection, scattered cells were observed throughout the bulb, including in the glomerular layer (Figure 1E). After reaching the appropriate layer, the morphology of GFP^+ ^cells changed, with cells transforming into more mature OB interneurons including mainly GC and less numerous PGCs.

The use of lentiviral vectors precludes precise birth-dating of newborn neurons. Nonetheless, from morphological and physiological points of view, the population of neuroblasts studied was homogeneous during the course of our analysis (around 10 days). We used the measurement of input membrane resistance (IR) as an index of cell maturation [31] and recorded GC in addition to RMS neuroblasts for comparison. In the RMS, we did not find a correlation between the IR and the number of dpi (n = 15 from 11 slices; *P *= 0.36; Additional file 3A), while a significant decrease in IR over time (*P *= 0.05) was seen for GFP^+ ^cells recorded in the GCL (n = 21 from 19 slices; Additional file 3B), consistent with the expected maturation of newborn GCs.

### Efficacy of a short hairpin RNA for NKCC1

To test the potential effects of NKCC1 on neuroblast migration and their electrophysiological properties, we used two different strategies. The first consisted of the pharmacological blockade of NKCC1 using the broad-spectrum blocker bumetanide [32,33]. To check for potential unspecific effects of bumetanide, we repeated all our measurements in cells transduced with a well-characterized RNA interference sequence to knock down the expression of NKCC1 [21,23,34].

**Figure 2:**
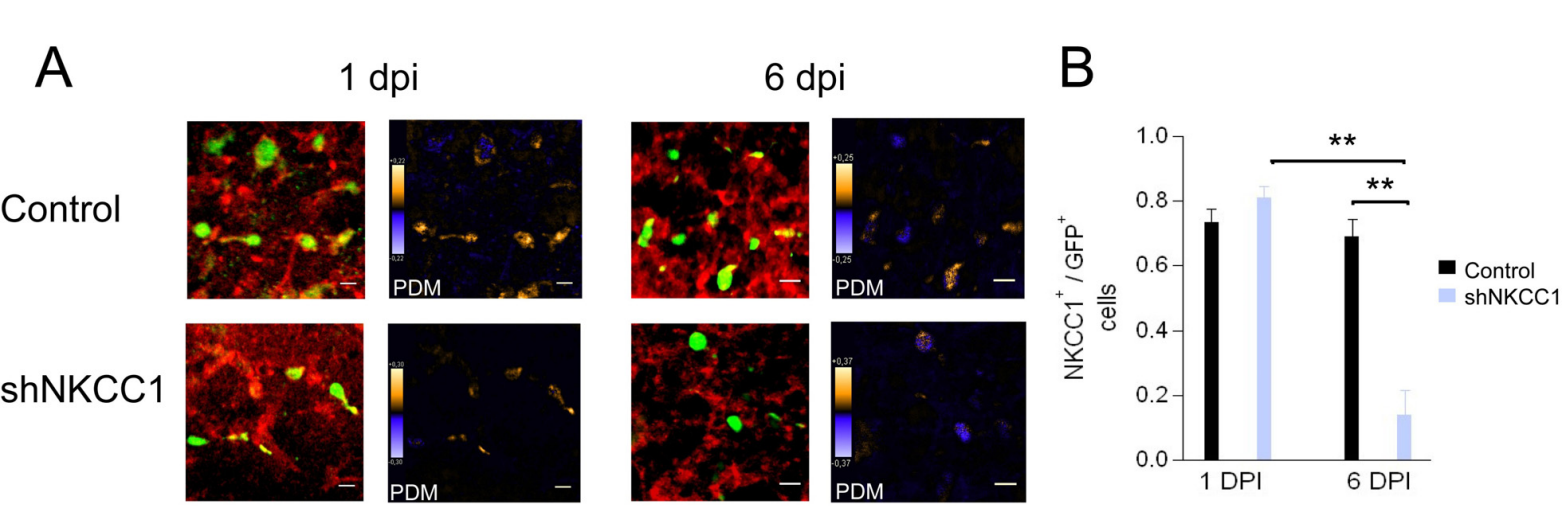
**Immunohistochemical staining for NKCC1 in organotypic cultured slices at 1 and 6 dpi**. **(A) **Upper panel: photomicrograph showing the immunolabeling for NKCC1 (red) and GFP (green) in RMS neuroblasts transduced with the control non-target sequence after 1 dpi (left) and 6 dpi (right). Each immunolabeling image is accompanied by an image corresponding to its analysis of the product of the differences from the mean (PDM), which shows the correlated areas in yellow and the segregated ones in blue. Lower panel: photomicrograph showing the immunolabeling for NKCC1 (red) and GFP^+ ^(green) in RMS neuroblasts transduced with the shNKCC1 sequence after 1 dpi (left) and 6 dpi (right), and their corresponding PDM analysis. Scale bars: 10 μm. **(B) **Normalized average (± standard error of the mean) number of neuroblasts showing correlated NKCC1 and GFP signals in cells transduced with the control and shNKCC1 sequences after 1 and 6 dpi. **P ≤ 0.01.

To validate the use of the short hairpin RNA (shRNA) sequence designed and characterized by Ge *et al. *[21], we performed immunohistochemical staining for NKCC1 on RMS neuroblasts. We compared the staining in cells transduced with either a lentiviral vector encoding GFP and a non-encoding control sequence (hereafter called the control) or a vector encoding GFP and the shRNA for NKCC1 (hereafter called shNKCC1). Two sets of experiments were done. First, we compared the number of neuroblasts coexpressing GFP and NKCC1 in slices transduced with either the control or the shNKCC1 encoding sequences after 1 and 6 dpi (Figure 2). Second, we compared the number of GFP^+^/NKCC1^+ ^cells in both the RMS and the GCL at 6 dpi transduced with either one of the sequences (Additional file 4). In both sets of experiments, we found a significant difference between groups using the Kruskal-Wallis test: values for each experiment were H = 33.82 (*P *= 0.1 × 10^-4^) and H = 38.13 (*P *= 0.1 × 10^-4^). Subsequent Mann-Whitney comparisons between the groups showed a significantly higher proportion of neuroblasts positive for NKCC1 in slices transduced with the control sequence compared to those transduced with shNKCC1 at 6 dpi: values changed in the first experiment from 0.69 ± 0.05 (n = 85 cells) to 0.13 ± 0.07 (n = 41 cells), *P *= 1 × 10^-3 ^and in the second experiment from 0.80 ± 0.09 (n = 58 cells) to 0.25 ± 0.11 (n = 63 cells), *P *= 1 × 10^-4^. In contrast, the proportion of NKCC1^+ ^GCs was small in control slices (0.22 ± 0.05; n = 27 cells) and did not significantly change in shNKCC1 slices at 6 dpi (0.05 ± 0.03; n = 17 cells; *P *= 0.09). In slices injected with the shNKCC1-encoding vector, we observed a significant reduction in the proportion of GFP^+^/NKCC1^+ ^neuroblasts when comparing results at 1 and 6 dpi (from 0.81 ± 0.03 to 0.13 ± 0.07; n = 22 and 41 cells; *P *= 1 × 10^-3^). Meanwhile, no difference was observed between 1 and 6 dpi in slices injected with the control vector (0.73 ± 0.03 and 0.69 ± 0.05; n = 67 and 85 cells; *P *= 0.25). The significance reported in this section takes into account the adjustment for *P*-values using the Bonferroni method.

Our immunohistochemical studies show that the shRNA interference strategy used in this study is efficient to reduce the expression of NKCC1 in neuroblasts, and coincides with its previously proven efficacy in the hippocampus [21,34] and the cortex [23]. Moreover, our results show that the expression of NKCC1 is reduced when the newborn cells leave the RMS to integrate the GCL.

### NKCC1 activity is necessary to maintain normal migration speed in RMS neuroblasts

**Figure 3:**
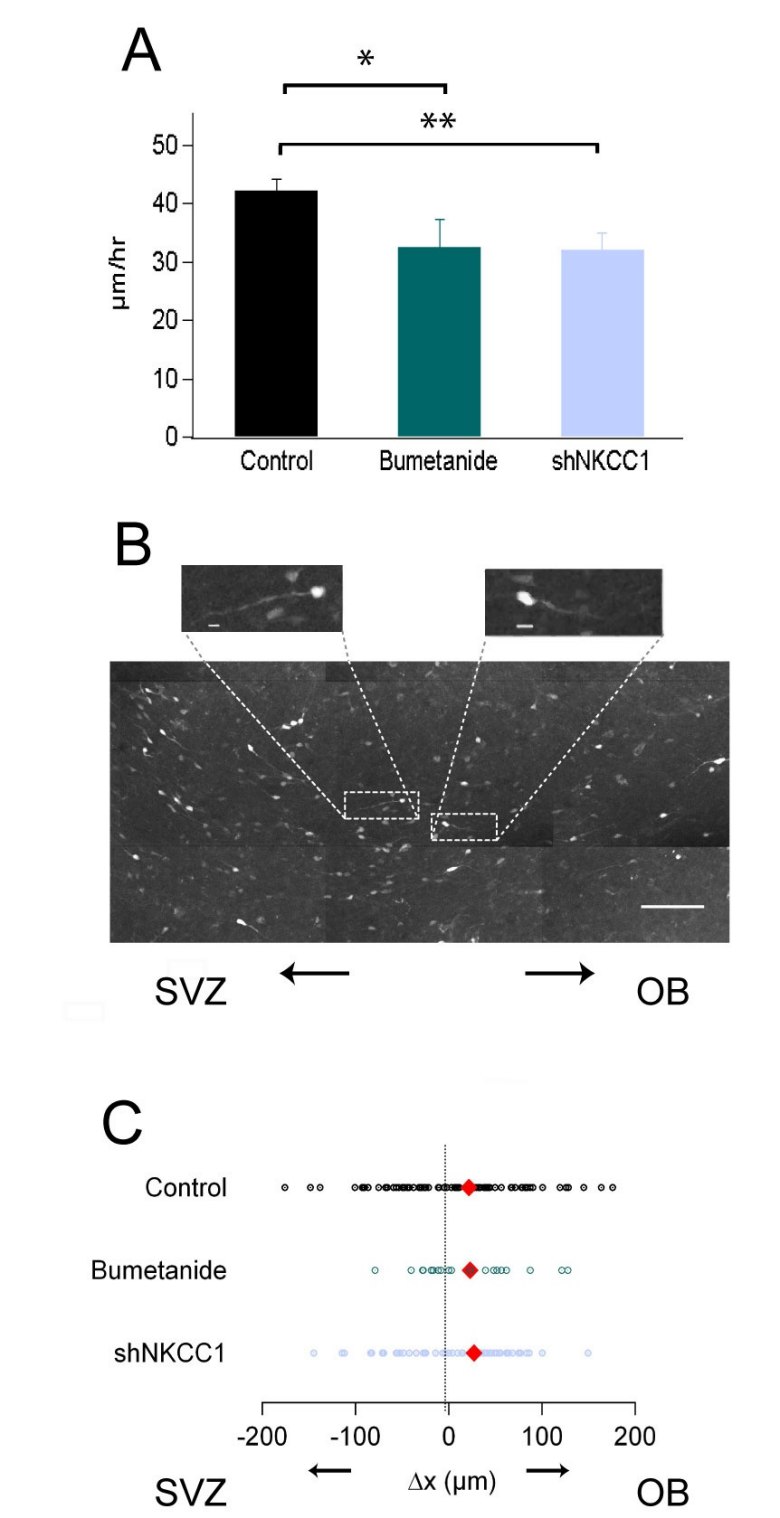
**The activity of NKCC1 regulates the migration speed of RMS neuroblasts**. **(A) **Plot showing the average migration speed of neuroblasts during a 3 hour recording session. Three groups are plotted: control (transduced with a control non-target shRNA sequence), bumetanide (transduced with a control non-target shRNA sequence and incubated in 10 μM bumetanide), and shNKCC1 (transduced with shNKCC1). **(B) **Photomicrograph of tangentially migrating GFP^+ ^neuroblasts in the RMS. The RMS was aligned horizontally so that changes along the x-axis represent displacement towards or away from the OB. Scale bar: 100 μm. Insets: detail of the directionality of the leading process in migrating neuroblasts. Scale bars: 10 μm. **(C) **Analysis of the migration directionality during the experimental session in the groups of cells described in (A). Displacement towards the SVZ is given a negative value whereas movements towards the OB are positive. Each point represents the total distance covered by one cell during the experimental session and the cells' initial position is at the origin. A red diamond represents the median value for each group. For this and the following figures *P < 0.05 and **P < 0.01.

We explored whether the diminution in the activity of NKCC1 could impair the migration of neuroblasts in the RMS. For this, we compared the tangential migration of cells transduced with the non-target shRNA-GFP (control) against control cells in slices incubated in 10 μM bumetanide 1 hour before and during the time lapse recording. A similar approach was employed with cells transduced with the shNKCC1-GFP virus. We calculated the average migration speed and the total displacement (∆X) along the horizontal axis of the RMS in 202 cells (from 9 slices) for controls; 61 cells (4 slices) incubated in bumetanide, and 83 cells (from 8 slices) transduced with shNKCC1, in 7 independent experiments. Control neuroblasts migrated with an average speed of 42.2 ± 2.0 μm/h. The migration speed became significantly slower in cells incubated in bumetanide (32.4 ± 4.7 μm/h; *P *= 0.02) or transduced with shNKCC1 (31.9 ± 3.1 μm/h, *P *= 0.007) (Figure 3A; Additional files 5 and 6).

Figure 3B illustrates RMS neuroblasts migrating back and forth with respect to the OB. To investigate whether NKCC1 was required for determining the net tendency of the cells to migrate towards the OB, we compared the total displacement of cells along the x-axis, and used it as an index for directionality (Figure 3C). The initial cell position was set to zero and the values of displacement were considered negative when cells moved towards the injection site (that is, the SVZ) and positive when cells headed towards the bulb. We found no significant differences in the values of ∆x between control cells and cells incubated in bumetanide or transduced with the shNKCC1 (*P *= 0.9 and 0.6, respectively).

### NKCC1 modulates the migration speed in a GABA_A_-independent manner

GABA acting on GABA_A _receptors reduces the migration of neuroblasts [12]. In order to test whether the effects observed by reducing the activity of NKCC1 were due to changes in the action of GABA_A _receptors, as a consequence of a potential drop in [Cl^-^]_i_, we analyzed the effects of blocking GABA_A _receptors with 10 μM gabazine in slices transduced with the control sequence, in slices transduced with the control sequence and treated with bumetanide, and in slices transduced with the shNKCC1 sequence.

**Figure 4:**
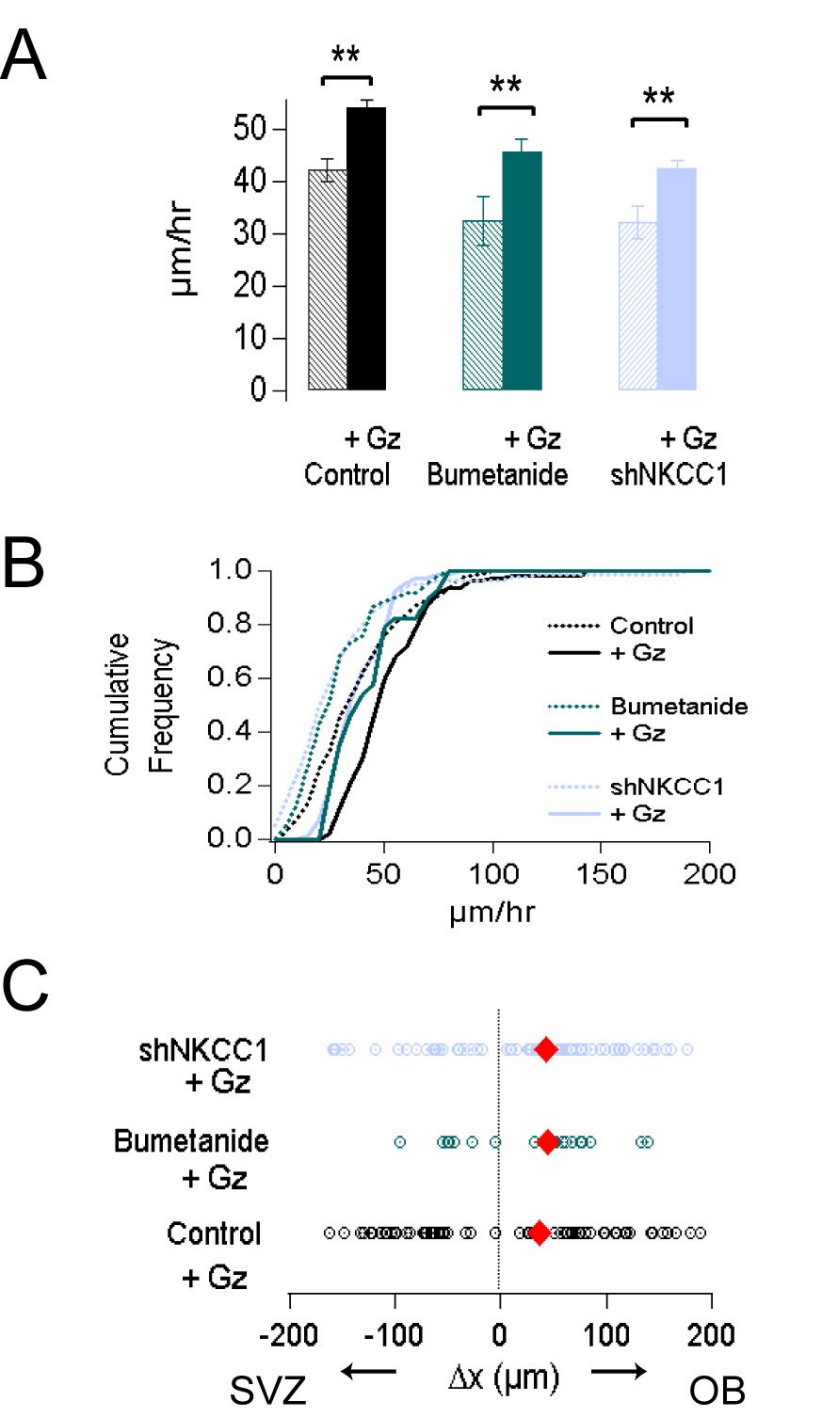
**NKCC1 affects migration of neuroblasts in a GABA**_**A**_**-independent manner**. **(A) **Plot of the average migration speed of neuroblasts from the control, bumetanide and shNKCC1 groups (hatched bars) and these groups after incubation in 10 μM gabazine (*+*Gz; solid bars). Error bars indicate standard error of the mean. **(B) **Normalized cumulative plot for the averaged migration speed in the groups displayed in (A). The shift to the leftward direction in the bumetanide and shNKCC1-transduced cells treated with gabazine compared to the gabazine treated control group indicates GABA_A_-receptor-independent effects (solid lines). **(C) **Analysis of the directionality of the migration during the experimental session in control, bumetanide-treated and shNKCC1-transduced cells incubated in gabazine. Analysis of directionality of migration as in Figure 3C.

We analyzed 69 cells transduced with the shNKCC1 sequence (from 4 slices), 43 cells treated with bumetanide (from 3 slices) and 112 cells transduced with the control sequence (from 5 slices); all were recorded in the presence of gabazine during the experiments. Our results were compared using a two-way ANOVA, which showed significant differences between the group injected with the control sequence, the group injected with control sequence and treated with bumetanide and the shNKCC1 sequence group (*P *= 1 × 10^-6^), as well as a significant effect of gabazine (*P *= 1 × 10^-6^). The differences between individual groups were then evaluated using a Tukey posthoc test. The comparisons show an increase in the average migration speed due to the GABA_A _receptor blockade in the three groups (Figure 4A). The average speed of control cells treated with gabazine was significantly faster than in the non-treated control group (53.9 ± 1.5 compared to 42.2 ± 2 μm/h; *P *= 1 × 10^-3^; Figure 4A). Gabazine also significantly accelerated the speed of migration when comparing bumetanide groups (from 34.4 ± 2.8 to 45.5 ± 2.5 μm/h; *P *= 0.01) and shNKCC1-treated cells (from 31.9 ± 3.1 to 42.5 ± 1.6 μm/h; *P *= 0.01). The cumulative distribution curve shifted to the right in all the groups treated with gabazine, compared with their respective controls (Figure 4B). The Kolmogorov-Smirnov test revealed no significant difference in the distribution of the average speed between control groups and bumetanide or shNKCC1 groups treated with gabazine (*P *= 0.99 and 0.13). Overall, the analysis shows a very significant effect of NKCC1 pharmacological and genetic blockade as well as a significant effect of GABA_A_-receptor blockers on the speed of migration of neuroblasts. Since the interaction between these two variables was not statistically significant (*P *= 0.78), we conclude that the role of NKCC1 on the maintenance of migration speed is independent of the modulation exerted by GABA_A _receptors.

To explore the effects of GABA_A _signaling on the directionality of the migration, we analyzed the displacement of the neuroblasts along the x-axis, as explained in the previous section. We did not observe any effect on direction after the blockade of GABA_A _receptors in any of the groups treated with gabazine - that is, control, bumetanide and shNKCC1 groups - compared to their respective non-treated groups (*P *= 0.8, 0.56 and 0.7, respectively; Figure 4C). Altogether, our results show that neither NKCC1 nor the GABA_A_-mediated activity are important in determining the directionality of RMS migrating neuroblasts.

Concerning the speed of migration, our experiments show that activation of GABA_A _receptors diminishes the migration speed in a way compatible with previous observations [12]. Moreover, the experiments performed in the presence of gabazine showed that the effects of NKCC1 on neuroblast migration do not depend on GABA_A _signaling.

### A tonic GABAergic current is active in both migrating neuroblasts and maturing GCs

Our whole-cell recordings show that migrating neuroblasts do not receive spontaneous synaptic inputs, confirming previous studies [13]. The lack of synaptic inputs in neuroblasts holds even after treatment with alpha-latrotoxin to trigger massive neurotransmitter release (data not shown, n = 3 cells from 3 slices) [35,36]. In contrast, newly formed GCs displayed spontaneous synaptic activity, which started at 6 dpi and significantly increased at 10 dpi.

**Figure 5:**
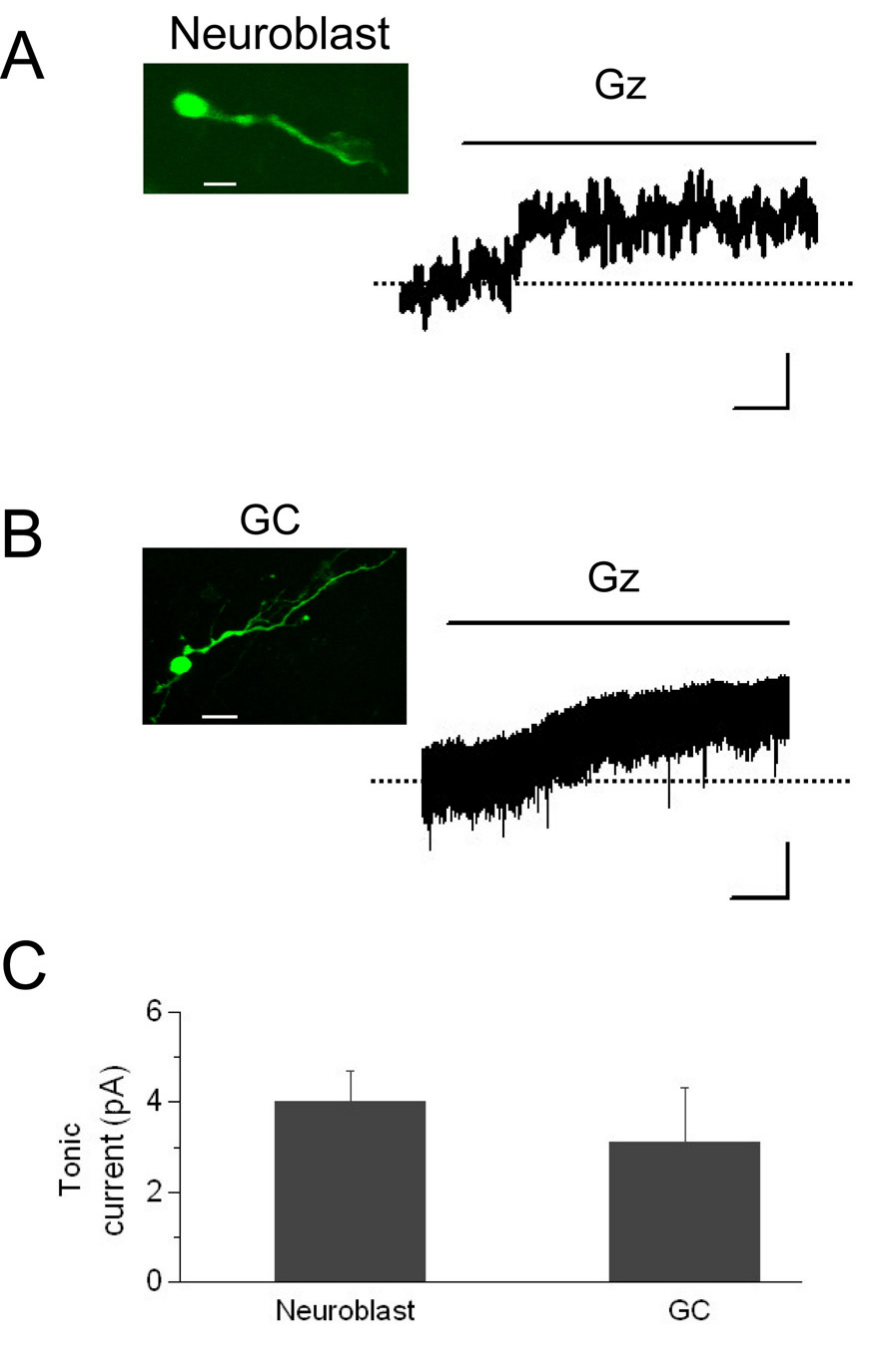
**Tonic GABA**_**A**_**-mediated currents in migrating RMS neuroblasts and maturing GCs**. **(A) **The photomicrograph shows the typical morphology of a migratory RMS neuroblast recorded at 8 dpi. Current trace shows the spontaneous activity recorded in a migratory neuroblast in the RMS voltage-clamped at -60 mV before and after adding 10 μM gabazine (Gz), revealing a tonic GABA_A _current. Scale: 5 pA and 5 s. **(B) **Photomicrograph showing the morphology of a GC recorded at 10 dpi. Example trace of a tonic GABA_A_-mediated current from GFP^+ ^cells recorded in the GCL. Scale: 10 pA and 20 s. **(C) **Plot of the average values for GABA_A_-mediated tonic currents in the two cell types studied. Photomicrograph scale bars are 10 μm in (A) and 20 μm in (B). Error bars indicate standard error of the mean.

Due to the lack of synaptic activity in neuroblasts, the action of GABA on the migration speed must depend on a tonic influence of this neurotransmitter. This mechanism has been suggested [12] but the tonic currents have never been recorded. Thus, we recorded the spontaneous activity of GFP^+ ^cells (5 to 11 dpi) held at -60 mV before and after applying the GABA_A _receptor blocker gabazine (10 μM). GFP^+ ^cells were either migrating neuroblasts located in the RMS (Figure 5A) or, for comparison, neurons located in the GCL (Figure 5B). In the GCL we paid particular attention to avoid recording from migrating GFP^+ ^cells, most likely representing newborn PGCs *en route *to the glomerular layer. Our results confirm the tonic action of GABA_A _receptors in neuroblasts and extend the observation to GCs. Moreover, they show that 10 μM gabazine, the same concentration used in our migration studies, efficiently blocks the tonic GABA_A_-mediated currents, which became evident from a diminution in holding current of 4.0 ± 0.7 pA in migrating neuroblasts (n = 10 cells from 9 slices; *P *= 9e-4, paired *t*-test) and of 3.1 ± 1.2 pA in GCs (n = 6 cells from 6 slices; *P *= 0.02, paired *t*-test) (Figure 5C) [37].

### NKCC1 controls E_GABA _in RMS neuroblasts but not in GCs

In order to understand the effects observed on neuroblast migration, we decided to determine whether and, if so, how the activity of NKCC1 affects the action of GABA_A _receptors and the overall excitability of the neuroblasts. For this we measured E_GABA _(the reversal potential for GABA_A _mediated responses) in RMS cells transduced with the control sequence and incubated in 10 μM bumetanide or in cells transduced with the shNKCC1 sequence. As a reference, we repeated our measurements in newborn cells at a more mature stage, that is, in GFP^+ ^GCs.

**Figure 6:**
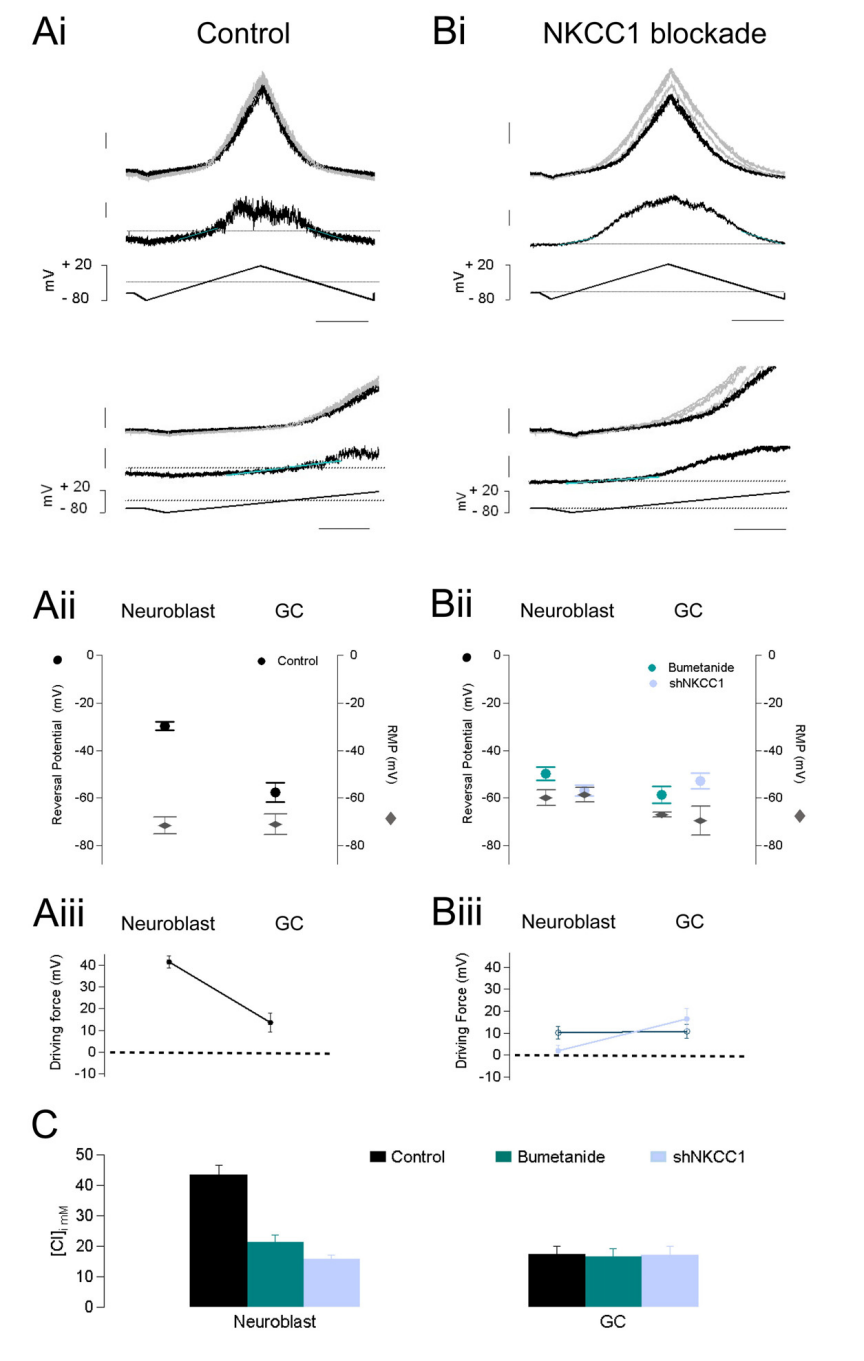
**NKCC1 controls the reversal potential of GABA**_**A **_**responses and resting membrane potential in migrating neuroblasts but not in GCs**. **(A) **Reversal potential of muscimol-induced currents and resting membrane potential (RMP) in cells injected with control shRNA. (Ai) Upper panel: current traces showing the responses to a voltage-ramp protocol (bottom trace) applied to a RMS neuroblast recorded with the gramicidin-perforated patch technique. The ramp was induced in the absence (black) and presence (gray) of muscimol puffed near the soma of the recorded cell. The reversal potential was determined by fitting a straight line (light blue) to the subtracted current (middle trace). Scale top to bottom: 50 pA, 20 pA, 200 ms. Lower panel: high magnification version of the trace in the upper panel. Scale: 100 pA, 40 pA, 100 ms. (Aii) Average reversal potentials (left axis; dots) of the muscimol responses in migrating neuroblasts and GCs. The RMP (diamonds) is indicated on the right axis of the same plot. (Aiii) Driving force for muscimol responses in control neuroblasts and GCs. **(B) **Reversal potential to muscimol-induced current and RMP in cells with impaired NKCC1 function. (Bi) Current traces elicited by a voltage ramp applied to a migrating neuroblast transduced with shNKCC1 (as described in (A)). Scale: 100 pA, 50 pA, 200 ms. Lower panel: higher magnification of the upper panel trace. Scale: 100 pA, 60 pA, 100 ms. (Bii) Reversal potential (left axis) and RMP (right axis) measured in migrating neuroblasts and GCs after incubating slices with 10 μM bumetanide (dark blue) and in cells transduced with shNKCC1 (light blue). (Biii) Driving force of GABA-induced currents, for each cell type, after bumetanide treatment (dark blue) or in shNKCC1-expressing cells (light blue). **(C) **Plot of calculated average +/- sem [Cl^-^]_i _values in migrating neuroblasts and maturing GCs in control conditions (black), after incubation with bumetanide (dark blue), and in cells transduced with shNKCC1 (light blue).

Gramicidin-perforated patch-clamp recordings were carried out to determine [Cl^-^]_i_, and the strength of GABA_A_-mediated responses. A voltage-ramp from -80 to +20 mV was applied in the presence and absence of a focal application of 10 μM muscimol (pressure ejected near the cell soma). The resulting currents were subtracted to calculate E_GABA _(Figure 6Ai,Bi). Our results show that NKCC1 is an important modulator of GABA action in neuroblasts, since the diminution of its activity by either of the means tested produced a significant hyperpolarization of E_GABA_. E_GABA _in migrating RMS neuroblasts shifted from -29.8 ± 1.7 mV (n = 6 cells from 6 slices) in control to -49.7 ± 2.8 mV in bumetanide (n = 9 from 6 slices; *P *= 0.01) and to -56.7 ± 2.3 mV in shNKCC1-treated neuroblasts (n = 8 from 7 slices; *P *= 4 × 10^-4^) (Figure 6Bii).

In contrast, new GCs had E_GABA _values significantly more hyperpolarized than neuroblasts in the control (-57.5 ± 4.1 mV; n = 13 from 13 slices; *P *= 6.9 × 10^-5^; Figure 6Aii) and this value was not sensitive to either bumetanide -58.6 ± 3.6 mV (n = 12 from 10 slices; *P *= 0.99) or shNKCC1 treatment (-52.8 ± 3.3 mV; n = 6 from 5 slices; *P *= 0.94) (Figure 6Bii).

We controlled for possible bias in our measurements due to the concentration of Cl^- ^in the patching solution. The comparison of E_GABA _values between independent groups of cells using pipette solutions containing either a low or a high [Cl^-^] (solutions 1 and 2 in Materials and methods) did not show any difference (*P *= 0.47) so the results were pooled. Furthermore, an additional control using immunohistochemical labeling for KCC2 showed that the hyperpolarization of E_GABA _in neuroblasts transduced with shNKCC1 is not due to a compensatory expression of KCC2, since no positive KCC2 labeling was observed in either GFP^+ ^neuroblasts or GFP^+ ^GCs (Additional file 7).

We used E_GABA _values to estimate [Cl^-^]_i _in both neuroblasts and GCs in the control and after treatments (Figure 6C). According to the effects on E_GABA_, the calculated values of [Cl^-^] decreased from 43.5 ± 3.1 mM in control neuroblasts to 21.4 ± 2.3 mM (*P *= 6.14 × 10^-5^) in bumetanide- and 15.9 ± 1.3 mM (*P *= 1.09 × 10^-5^) in shNKCC1-treated cells. In newly formed GCs the values of [Cl^-^] in bumetanide- (16.4 ± 2.6 mM; *P *= 0.99) and shNKCC1-treated cells (17.2 ± 2.8 mM; *P *= 1) were not significantly different from those in the control (17.5 ± 2.7 mM). The lack of effect of the treatments diminishing NKCC1 activity on GCs agrees with the low expression of NKCC1 observed in the immunohistochemical analysis (Additional file 4B,C).

The values of E_GABA _and [Cl^-^] were constant in each cell type for the duration of our study as no significant correlation between E_GABA _and dpi was observed in Spearman correlation tests (r = -0.11 for neuroblasts and r = -0.22 for GCs; Additional file 8).

### NKCC1 controls the resting membrane potential in RMS neuroblasts

To understand the net effect of the activation of conductance on a cell's membrane potential, it is useful to determine the value of the potential at rest. The resting membrane potential (RMP) was measured in the cell-attached mode as the equivalent of the reversal potential of K^+ ^conductances using isotonic intra- and extracellular concentrations of this ion [38]. We did verify that, in our system, this method was sensitive to predictable changes in RMP by measuring the reversal of K^+ ^currents before and after raising the extracellular [K^+^] to 10 mM. This manipulation produced an average 3.4 ± 1.6 mV depolarization in the four cells tested.

We then measured the RMP in migrating neuroblasts and GCs in control slices. Remarkably, we did not find significant differences in RMP values between these cell groups: -71.5 ± 3.6 mV (n = 6 from 5 slices) and -71.0 ± 4.4 mV (n = 6 from 6 slices; *P *= 0.93, *t*-test), respectively. Nonetheless, the comparison of RMP in neuroblasts treated with bumetanide or shNKCC1 showed a depolarization due to the diminution of NKCC1 activity. The measured values were -59.8 ± 3.2 mV (n = 6 from 5 slices; *P *= 0.07) for bumetanide and -58.7 ± 2.9 mV (n = 9 from 5 slices; *P *= 0.02) in cells expressing shNKCC1 (Additional file 9). In contrast, in GCs the diminution of NKCC1 activity did not produce significant changes in RMP compared to control; values of -67.0 ± 1.0 mV (n = 2 from 2 slices; *P *= 0.90) in bumetanide- and -69.4 ± 6.1 mV (n = 5 from 5 slices; *P *= 0.97) in shNKCC1-treated cells.

We used the E_GABA _values from muscimol-mediated currents and the RMP values to calculate the driving force for GABA-mediated responses. In neuroblasts, both the hyperpolarization of E_GABA _as well as the depolarization of about 11 mV in their RMP, observed in bumetanide and shNKCC1 groups, resulted in an important decrease in the driving force for GABA responses, with the strongest effects on the shNKCC1-treated group. The calculated driving force value in control neuroblasts (41.7 ± 2.8 mV) was significantly diminished to 10.2 ± 2.9 mV (*P *= 2.84 × 10^-5^) in bumetanide- and to 2.0 ± 2.6 mV (*P *= 1.03 × 10^-5^) in shNKCC1-treated cells. Moreover, as expected from the lack of effect of the diminution of NKCC1 activity on both E_GABA _and RMP in GCs, we did not observe any effect on the driving force between control GCs (13.5 ± 4.2 mV) and the bumetanide- (10.81 ± 3.6 mV; *P *= 0.98) or shNKCC1-treated groups (16.6 ± 4.8 mV; *P *= 0.99) (Figure 6Aiii).

A comparison between cell types - neuroblasts versus GCs - in the control condition shows a significant decrease in the driving force for GABA_A_-mediated responses in newborn interneurons when reaching the bulb (*P *= 5.28 × 10^-5^; Figure 6Aiii,Biii). Nonetheless, GABA maintained a depolarizing action on both cell types. Our results indicate that NKCC1 expression is diminished in GCs from the time of their arrival at the OB. Therefore, in sharp contrast to neuroblasts, neither the action of GABA nor the values of RMP depend on NKCC1 activity in developing GCs.

Finally, we verified whether gabazine was able to change the RMP in neuroblasts, as would be expected from the measured driving forces for muscimol responses. In control cells, gabazine induced a 2.3 ± 0.8 mV (n = 3 cells) hyperpolarization of the RMP while only a 0.45 ± 0.04 mV hyperpolarizing shift was observed in shNKCC1 neuroblasts (n = 2). The lesser effect of gabazine on RMP in shNKCC1 cells supports the diminished driving force for muscimol observed in these cells. Moreover, the lesser effect on RMP observed in shNKCC1 cells might be related to the decreased effect of gabazine on the acceleration of neuroblast migration in the bumetanide and shNKCC1 cells (Figure 4).

## Discussion

In the present study we characterize the role of NKCC1 on the regulation of the migration and excitability of migratory neuroblasts and developing OB interneurons in early postnatal tissue. We found that NKCC1 activity is important for the neuroblasts to maintain a normal migratory speed since two independent manipulations employed to impair NKCC1 function, pharmacological blockade with bumetanide and transduction with shNKCC1, produced a diminution of the migration speed (Figure 3A). Meanwhile, the manipulation of NKCC1 did not affect the directionality of migration (Figure 3C), suggesting that these two aspects are regulated by different mechanisms.

We performed electrophysiological measurements to determine the effects of NKCC1 on the excitability of migratory neuroblasts. Our results show that, similar to other developing neurons [19-21], the activity of NKCC1 maintains a high concentration of [Cl^-^]_i _in RMS neuroblasts (Figure 6Bii). In addition, we demonstrate that the activity of this cotransporter is necessary to keep a hyperpolarized RMP in neuroblasts (Figure 6Bii), and thus to control the resting excitability in these cells in a manner independent of the regulation of Cl^- ^fluxes initiated by neurotransmission. This result supports previous studies showing modifications of RMP and neuronal excitability after manipulation of Cl^- ^cotransporters in different types of neurons [20,39-41]. It is likely that changes in the ionic concentration of Na^+ ^contributed by the activity of Cl^- ^cotransporters alter the homeostasis and resting potential of the cells. Along these lines, manipulations depleting the internal concentration of this ion have been shown to depolarize the RMP of neonatal neurons [39]. Possible explanations relating the alterations in Na^+ ^fluxes and modulations of RMP could involve diminished electrogenic activity of ionic carriers such as Na^+^/K^+ ^ATPase, thus producing a depolarization of the RMP [42], or changes in the magnitude of K^+ ^conductances, very prominent in the RMS neuroblasts [13]. The exact mechanisms explaining the significant depolarization of RMP observed in the RMS neuroblasts after the blockade of NKCC1 is beyond the scope of the present study. Nevertheless, the alteration of RMP after the blockade of Cl^- ^cotransporters observed for the first time in migratory neuroblasts in the present study and in other cell types in previous reports (see above) suggests a relevant role for NKCC1 in the control of resting excitability in developing neuronal cells.

Our results show that the blockade of GABA_A _receptors accelerates the migration speed of the neuroblasts in the RMS. These observations are in agreement with previous reports showing that tonically released GABA acts on GABA_A _receptors to diminish the velocity of migration in RMS neuroblasts [12,43,44], as well as in the cortex [45]. In our study, the speed of migration was accelerated by GABA_A _pharmacological blockade in both neuroblasts transduced with the control sequence and those treated with bumetanide or transduced with shNKCC1. This is in agreement with the depolarizing driving force for GABA measured in all groups of cells (Figure 6Aiii,Biii), as well as with the hyperpolarization of RMP values observed after treating the cells with gabazine. The statistical analysis of the effects of blockers of GABA_A _receptors and NKCC1 shows a lack of interaction between these two mechanisms. Thus, our results strongly support the notion that the motogenic action of NKCC1 does not depend on the activity of GABA_A _receptors. It is very likely that the effects of NKCC1 blockade on neuroblast migration are accounted for by the sustained depolarization of the RMP observed in cells with impaired NKCC1 function. In support of this hypothesis, a similar study by Bolteus and Bordey [12] shows that depolarization induced by KCl treatment diminishes the speed of RMS neuroblast migration. In that study, the diminution of speed induced by depolarization was only partially reverted, but not abolished, by the pharmacological blockade of GABA_A _receptors with 100 μM bicuculline. Thus, our results, together with previous findings, show that the migration of neuroblasts is very sensitive to manipulations affecting the resting membrane potential, therefore suggesting that the relationship between the membrane potential and the speed of migration might be more important than previously thought.

The precise way in which external and internal processes regulate neuroblast migration in the RMS remains unclear. Modifying bulbar neuronal activity has little effect on tangential migration. For instance, anosmia [46], naris occlusion [47], or surgical disruption of the RMS [48] failed to alter neuroblast migration. In contrast, migration in the RMS is highly sensitive to brain hyperexcitability, associated with status epilepticus [49], or to brain injury, which accelerates the speed of migration [49,50]. Further studies are needed to understand how these external signals can change neuroblast excitability and adjust their rate of integration into the OB circuits or injured brain areas. Considering the important role of NKCC1 in the integration of external signaling and resting excitability, further investigations should determine whether pathological brain activity can impact the function of this Cl^- ^cotransporter, particularly since previous studies have shown that NKCC1 function is sensitive to activity-dependent modulation [51,52]. In this regard, a recent study showed that the pharmacological blockade of NKCC1 diminishes the proliferation of glial precursors induced by ischemia in the cortex [53].

### NKCC1 is inactivated during the transition from migrating RMS neuroblasts to GCs

In the present study we used organotypic slice cultures to compare the role of NKCC1 on the excitability of neuroblasts and in maturing newborn neurons. The use of this model seems particularly appropriate for the present study since both the neuroblast migration and GC integration occur in the organotypic control, in a similar way to those reported from acute models [12,31]. First, the speed of migration in our system is remarkably similar to previous values measured in acute slices [12]. Moreover, the labeled precursors evolve in the organotypic culture, first as a contingent of neuroblasts migrating in the RMS to increasingly populate the OB. Once in the GCL, the cells change their morphology and show functional maturation (Figure 1; Additional file 3), including the development of synaptic activity. A similar system has been previously used to study the control of differentiation in dopaminergic cells [54].

Our results show that the early maturation of OB interneurons, at least when considering GCs, is paralleled by changes in both GABAergic signaling and NKCC1 function. After reaching the bulb, the neural precursors acquire a mature morphology and establish synaptic interactions with pre-existing neurons, some of which are GABAergic [55]. Our results show that GABA action evolves from a non-synaptic, temporally and spatially diffuse tonic influence in the RMS to include synaptic contacts on GCs during the first weeks of development. The tonic influence of GABA was preserved in GCs during the entire duration of our study, regardless of the development of synaptic contacts. These results are in agreement with a previous report showing that, in early postnatal tissue, GABA produced a depolarizing influence, capable of shunting the firing activity on GCs [56]. Correspondingly, we detected a small tonic current and a slightly positive driving force for Cl^- ^in GCs (Figures 5 and 6A iii). Slightly positive driving forces for GABA are frequently observed in immature interneurons [57-59] and are believed to favor neuronal development [14].

The manipulations affecting the activity of NKCC1 showed that the strong depolarizing action of GABA in RMS neuroblasts largely depends on this Cl^- ^transport system. Meanwhile, in GCs, GABA exerted a much weaker depolarizing driving force that was not affected by the manipulations blocking the activity of NKCC1. These results are confirmed by the immunohistochemical data showing a much higher proportion of neuroblasts expressing NKCC1 compared to GCs in slices injected with the control sequence, and a significant drop of NKCC1^+ ^neuroblasts but not GCs in cells transduced with shNKCC1 (Additional file 4). In accordance with the functional and anatomical evidence of low expression of NKCC1 on GCs, we failed to find any effect of the manipulation of this Cl^- ^transporter on the development of GC dendrite projections (data not shown). Thus, it seems unlikely that GC synaptic connectivity in the OB is shaped by NKCC1, in contrast to the dentate gyrus [21].

Wang and colleagues [56] showed that, after the second postnatal week, GABA hyperpolarizes the GC, held at -60 mV, at the time that KCC2 mRNA appears. We observed a strong hyperpolarization of E_GABA _when GFP^+ ^cells reached the GCL. However, GABA action did not become hyperpolarizing during our study, possibly owing to delays in this process due to *in vitro *conditions, or because our recorded cells were young and did not yet express KCC2 (Additional file 7B). Several reports have shown that KCC2 mRNA expression is abundant in mature neurons, whilst negligible expression was detected in neuronal progenitors [17,60]. It is conceivable that, at later stages of maturation, KCC2 is involved in the synaptic connectivity of GCs, as it is in other systems [40,61]. Nonetheless, our results indicate that the first significant decrease in [Cl^-^]_i _in GCs, and therefore the simultaneous hyperpolarization of E_GABA_, results from a functional down-regulation of NKCC1.

Altogether our data suggest that variations in NKCC1 activity can efficiently control various steps of OB postnatal neurogenesis. Accordingly, we show that an important change in the functional expression of this cotransporter accompanies the developmental transition from neuroblasts to developing GCs. NKCC1 is highly active in the RMS precursor cells and its functional expression drops drastically in the newborn GCs, greatly affecting the action of GABAergic signaling on this early period of functional integration. Concerning the neuroblasts in the RMS, our results show, for the first time, an important role of NKCC1 in the control of neuroblast excitability. Our electrical determinations indicate that NKCC1 controls at least two different aspects of neuroblast activity. The first concerns the control of [Cl^-^]_i _and therefore the integration of the most prominent neurotransmitter signaling system in the RMS, GABA_A_-receptor-mediated activity [12,13,31,43,62,63]. The second aspect concerns the regulation of intrinsic excitability and depends on the control of the RMP. Thus, our study suggests that NKCC1 integrates relevant external signaling and the intrinsic neuronal excitability to finely tune the migration speed of RMS neuronal precursors *en route *to the OB.

## Materials and methods

### Culture preparation

Postnatal day 7 C57BL/6J mouse pups (Janvier, Le Genest Saint Isle, France)) were decapitated and the brains removed and placed into ice-cold artificial cerebrospinal fluid (ACSF) containing (in mM) 124 NaCl, 3 KCl, 1.3 MgSO_4_, 26 NaHCO_3_, 1.25 NaH_2_PO_4_, 10 glucose and 2 CaCl_2_, saturated with 95% O_2_/5% CO_2_. Sagittal forebrain slices, 300 μm thick, containing the migratory pathway from the SVZ-RMS and OB were prepared with a vibratome (Leica VT1200). All experimental procedures were performed in accordance with the Charter of Fundamental Rights of the European Union (2000/C 364/01), the European Communities Council Directive of 24 November 1986 (86/609/EEC), and European Union guidelines. They were reviewed and approved by our Institutional Animal Welfare Committee.

Each brain slice was transferred to a Millicell-CM Culture Plate Insert (Millipore PICM ORG 50; Billerica, MA, USA ), the excess ACSF was removed and the insert was placed in a 35-mm Petri dish containing 1 ml of medium, as described by Stoppini and colleagues [64]. Culture medium consisted of 46% MEM (31095-029, Invitrogen, Paisley, UK), 25% HBSS (24020-091, Invitrogen), 25% horse serum, 20 nM HEPES, 6 mg/ml D-glucose and gentamycin (Invitrogen, Paisley, UK). The slices were incubated at 37°C in a humid atmosphere of 5% CO_2. _The medium was changed three times per week.

### Lentiviral vectors

We used lentiviral vectors created using the PTRIPDU3 method to express GFP in neural precursors and neuroblasts [65]. The first vector was constructed such that GFP expression was under the control of the PGK promoter. For the second vector, a previously published shRNA sequence for NKCC1, shNKCC1-1 (5'ACACACTTGTCCTGGGATT3'), was introduced under the control of the U6 promoter [21]. This vector was similar to the first, also containing a sequence to induce GFP expression, but under the control of the CMV promoter. As a control, we constructed a third vector in which the shRNA sequence was replaced with a non-target scrambled sequence (5'GCCAGATTTCTCAGGTGATAA3'). Twenty-four hours after culturing the slices, 270 pg of p24 from one of the constructions was injected in the SVZ, in a volume of 18 nl.

### Electrophysiology

GFP^+ ^RMS neuroblasts and olfactory bulb GCs, at 3 to 11 dpi, were recorded using the patch-clamp technique in different configurations as detailed below. For data acquisition and electrical control an EPC-10 amplifier (Heka Elektronik, Lambrecht/Pfalz, Germany ) was used. Before the recordings, a piece of Millicell membrane containing the slice was cut out and transferred to a chamber. During the recording session the slices were continuously perfused at a rate of 1.5 ml/minute with extracellular solution containing (in mM) 124 NaCl, 3 KCl, 2.4 CaCl_2_, 1 MgCl_2_, 25 NaHCO_3_, 1.25 NaH_2_PO_4_, and 10 glucose. The extracellular solution was heated to 35°C at the entrance of the chamber (inline heater TC-324B, Warner Instruments, Inc; Hamden, CT, USA). Glass pipettes with a resistance of 6 to 7 MΩ were used for the recordings.

The reversal potential of GABA_A_-receptor-mediated currents was determined using the perforated patch-clamp configuration with gramicidin - a cation-specific channel - in order to obtain voltage control of the cell while leaving [Cl^-^]_i _intact [66]. For perforated-patch recordings one of the following patching solutions was used in each experiment: (1) 150 mM KCl, 10 mM Hepes; or (2) 150 mM KGluconate, 10 mM Hepes, both at pH 7.5. A stock of 1 mg/ml of gramicidin was freshly prepared on the day of the experiment and a final concentration of 2 μg/ml was added to the patching solution. The pipette tip was dipped in gramicidin-free patching solution before backfilling it with the gramicidin-containing solution. A giga-seal was established with the cell membrane and the recording session began when a stable access resistance between 50 and 70 MΩ was obtained.

To determine the reversal potential of the currents elicited by the GABA_A _receptor agonist muscimol, a 1 s voltage-ramp from -80 to +20 mV and back to -80 mV was applied to cells recorded in the perforated configuration. Muscimol (10 μM) was pressure ejected (10 to 20 psi) near the cell membrane during the ramp. At least three ramps were applied and averaged before and during muscimol application. The averaged currents were subtracted and the resulting trace was used to measure the reversal potential of the muscimol-triggered current. Reversal potentials were determined by linear fitting of the current trace using Igor (Wavemetrics, Inc; Lake Oswego, OR, USA), whereas [Cl^-^]_i _was estimated from the Nernst equation. Repeated agonist application can influence the apparent Cl^- ^reversal potential (E_GABA_), particularly in immature neurons in which Cl^- ^regulation mechanisms are less effective [67]. Therefore, we ensured that the interval between agonist applications was sufficiently long (20 s) to avoid shifts in E_GABA_.

To determine the cells' RMP we used the method described by Verheugen *et al. *[38]. Briefly, we kept the cells in the cell-attached mode at -60 mV and applied a voltage-ramp from -100 to +200 mV to activate a voltage-dependent K^+ ^conductance. Patching solutions 1 or 2, described above, were used in the absence of gramicidin for these determinations. Both solutions have a concentration of 150 mM K^+^; therefore, the reversal potential of the K^+ ^conductance was assumed to be equal to the RMP. The difference between average RMP values and the reversal potential of the muscimol-induced current values was used to determine the driving force for GABA in each condition and cell type.

Tonic currents were recorded in the whole cell mode at -60 mV holding potential. For these recordings solution 1 was supplemented with 2 mM ATPMg, 0.2 mM GTPNa and 0.2 mM EGTA. We recorded the spontaneous activity of the cells held at -60 mV for at least 2 minutes in normal ACSF before applying 10 μM gabazine to the bath, and then recorded spontaneous activity for 5 minutes after the application of the drug. To measure the tonic GABA_A _current, we assessed the average holding current necessary to maintain the voltage at -60 mV, during 10 s in control ACSF, starting 40 s before application of gabazine, and 30 s after its application in the bath. Bicuculline blocks SK-type K^+ ^channels [68] and may affect the measurement of tonic current. Thus, gabazine is the drug of choice for studying tonic GABA conductance. For one group of cells, GABA_A_-dependent tonic currents were determined in the cells recorded first in the perforated-patch configuration and subsequently transformed into whole-cell mode by gentle suction within the recording pipette (n = 6 neuroblasts and 3 GCs). A second group was recorded in whole cell mode in the absence of gramicidin on the intracellular pipette (n = 4 neuroblasts and 3 GCs). No significant differences were observed between the values obtained with either method, neither for the RMS neuroblasts (*P *= 0.57) nor for the GCs (*P *= 0.51); thus, the results were pooled.

Only the cells maintaining a stable access resistance (less than 20% variation during the experiment) were analyzed. The series resistance values were between 10 and 25 MΩ and no compensation was applied. The spontaneous currents were filtered at 2.9 KHz with a low-pass Bessel filter and were sampled at 8.33 KHz. To analyze the passive properties of cells, the currents generated by a 20 ms, 20 mV hyperpolarizing pulse were filtered at 5 kHz and sampled at 10 kHz. All values were corrected for the measured liquid junction potential (-2 and -8 mV for solutions 1 and 2, respectively).

### Drugs

All drugs were purchased from Sigma-Aldrich and used at the following concentrations: 10 μM bumetanide was used to block NKCC1 activity. In all the experiments using bumetanide the slices were pre-incubated for 1 h in 10 μM bumetanide and the same concentration was present during our electrophysiological and imaging recordings (Sigma-Aldrich, Saint-Quentin Fallavier, France). 10 μM gabazine and 10 μM muscimol were used as antagonist and agonist of GABA_A _receptors, respectively, and 10 nM alpha-latrotoxin was used to trigger neurotransmitter release.

### Immunohistochemistry

Organotypic brain slices were fixed in 4% paraformaldehyde before being cryoprotected in 12.5% sucrose, 5% glycerol for 2 h at 4°C and incubated overnight in 25% sucrose, 10% glycerol. Slices were rapidly frozen and thawed three times over liquid N_2 _vapor, washed in PBS before a second fixation in paraformaldehyde for 15 minutes, and incubated for 2 h in 10%, NGS 0.1% Triton blocking solution at room temperature. For immunolabeling of chloride cotransporters, slices previously injected with one of the lentiviral vectors were then incubated at 1 or 6 dpi in the primary antibody for NKCC1 (1:200; T4, Developmental Studies Hybridoma Bank) or at 6 dpi in the antibody for KCC2 (1:200; 07-432, Upstate, Billerica, MA, USA) mixed with the anti-GFP (1:1,000; 06-896, Upstate). A different set of organotypic slices at 6 div were incubated in the following primary antibodies: neuronal nuclei marker NeuN (1:200; MAB377, Chemicon, Billerica, MA, USA; n = 3), DCX (1:1,000; Ab18723, Abcam, Cambridge, UK; n = 3), GABA (1:1,000; A2052, Sigma; n = 3) or TH (1:4,000; 22941, ImmunoStar, Hudson, Wisconsin, USA; n = 3). All primary antibodies were diluted in blocking solution and incubated for 48 h at 4°C, followed by washes in PBS. Then, slices were incubated for 2 h in an alexa-conjugated secondary antibody (Alexa Fluor 568 (1:750) for the chloride transporters and Alexa Fluor 488 (1:800) for the rest of the proteins; both from Molecular Probes, Paisley, UK) at room temperature and washed in PBS. NeuN, DCX, GABA and TH stained cultures were then incubated in DAPI for 15 minutes at room temperature and washed in PBS. All the slices were then mounted in ProLong Gold antifade (P36930, Molecular Probes). For analysis, images of 0.5 to 1 μm thick were acquired either in an upright microscope (Zeiss), equipped with a confocal spinning disk unit (Andor, Belfast, UK) or in an inverted microscope (Axioscope FS, Zeiss), equipped with a structured illumination device (Apotome, Zeiss).

To determine the number of GFP^+ ^cells in the field that were also positive for NKCC1, we used the Intensity Correlation Analysis package of ImageJ [69]. The region analyzed was first defined by the limits of the GFP signal of a cell in the z-axis. Signal was considered a cell when the morphology corresponded to a round or elliptical cell body of 6 to12 μm with at least one projection. We considered as double labeled those cells showing a positive intensity correlation quotient for both GFP and alexa 568 signals. The number of labeled cells per field (313 × 313 μm) was determined in three independent images for each region of interest (RMS and GCL), and the results were normalized to the total number of GFP^+ ^cells analyzed in each image and then averaged (Figure 2; Additional file 4).

### Time-lapse videomicroscopy

The Petri dish containing the cultured slice was transferred to a closed chamber with the temperature control adjusted to maintain the medium at 36°C (DH40i, Warner Instruments, Inc.). During the entire time-lapse session, the tissue was oxygenated using a mixture of 95% O_2_/5% CO_2_. To prevent evaporation of the culture medium, the internal environment of the chamber was continuously humidified (Humidifying module warmer HMW-1, Warner Instruments, Inc.). Mosaic images of the entire RMS were re-constructed from individual frames (x-, y- and z-axis), acquired every 20 minutes during a period of 3 h. We used an inverted fluorescence microscope (Axioscope FS, Zeiss) equipped with a 20× long-distance objective (LD plan Neofluar, Zeiss). For analysis, a maximal projection of the different planes taken along the z-axis was obtained for each time-lapse. Individual RMS neuroblasts could be identified and their trajectories were tracked using Metamorph (Molecular Devices). Two migration variables were analyzed, the speed and displacement along the x-axis, corresponding to the tangential displacement in the RMS, provided that the rostro-caudal axis of the RMS was positioned parallel to the x-axis (Figure 3B,C). Delta x (∆x), the total displacement of a cell along the rostro-caudal axis of the RMS during an experimental session, was used as an index of the direction of migration in the RMS-OB pathway.

### Statistical analysis

All results are expressed as averages ± standard error of the mean. Experiments involving the comparison of a control and an experimental group were analyzed using the Student's *t*-test. For multiple comparisons either one- or two-way ANOVA were used with the Tukey method as a post-hoc test; the use of each is detailed in the Results section. For multiple comparisons of the immunohistochemical data Kruskal-Wallis and Mann-Whitney methods were used; in these cases significance was evaluated based on the Bonferroni adjustment of *P*-values. Differences were considered significant for values of *P *< 0.05. Distributions were compared using the Kolmogorov-Smirnov test in the R program [70].

## Abbreviations

ACSF: artificial cerebrospinal fluid; DCX: doublecortin; div: days *in vitro*; dpi: days post-injection; GABA: gamma-aminobutyric acid; GC: granule cell; GCL: granule cell layer; GFP: green fluorescent protein; IR: input membrane resistance; KCC2: K^+^-Cl^-^; NKCC1: Na^+^-K^+^-2Cl^-^; OB: olfactory bulb; PBS: phosphate-buffered saline; PGC: periglomerular cell; RMP: resting membrane potential; RMS: rostral migratory stream; shRNA: short hairpin RNA; SVZ: subventricular zone; TH: tyrosine hydroxylase.

## Competing interests

The authors declare that they have no competing interests.

## Authors' contributions

The experimental work of this study was achieved at the laboratory for Perception and Memory, at the Pasteur Institute in Paris. S-MG and PM-L conceived and designed the experiments. K-M produced the viral vectors, organotypic cultures, immunohistochemical staining and corrected the English in the manuscript. S-MG performed the electrophysiological and migration experiments as well as the data analysis and interpretation. S-MG and PM-L wrote the manuscript.

## Supplementary Material

Additional file 1 **Figure S1**. Immunohistochemical staining for doublecortin (DCX) and neuronal nuclei marker (NeuN) in 6-div organotypic cultures. **(A) **Low magnification image (10×) from a sagittal organotypic slice stained with DAPI (blue) depicting the cellular nuclei in the rostral migratory stream (RMS), as delimited by dashed lines. **(B) **DCX (green) and DAPI labeling in the RMS elbow area. Inset: approximate location of the area shown in (C). **(C) **High magnification detail of the RMS double stained for DCX and DAPI. **(D) **Low magnification image showing the distribution of NeuN-expressing cells (green) in the granule cell layer (GCL) and absence of positive cells in the RMS in a sagittal organotypic slice of the OB. Outlined by dashed lines, the borders of the OB and the accessory OB are depicted for reference. **(E) **High magnification detail of the GCL region and OB RMS showing NeuN-expressing cells in the former and the cellular nuclei stained with DAPI. **(F) **Higher magnification detail of the OB RMS and GCL double stained for DAPI and NeuN. Scale bars: 250 μm for (A,E), 200 μm for (B,F), 20 μm for (C), and 500 μm for (D). Click here for file

Additional file 2 **Figure S2**. Immunohistochemical staining for GABA and tyrosine hydroxylase (TH) in 6-div organotypic cultures. **(A,B) **Photomicrograph showing GABAergic cells (green) and cellular nuclei co-stained with DAPI (blue) in the RMS (A), and in the GCL regions (B). **(C) **Photomicrograph showing the presence of TH-positive cells (green) surrounding a glomerulus in the OB, in an organotypic slice co-stained with DAPI. Inset: approximate location of the region magnified in (Ci). (Ci) High magnification photomicrograph showing a TH-positive cell costained with DAPI in an OB glomerulus. **(D) **Photomicrograph showing TH-expressing cells in the OB of an organotypic slice. Inset: approximate location of the region depicted in (Di). (Di) High magnification photomicrograph of a TH-expressing cell double labeled with DAPI in the OB. Scale bars: 50 μm for (A), 20 μm for (B), 100 μm in (C,D), 20 μm in (Ci,Di). Click here for file

Additional file 3 **Figure S3**. Functional maturation of recorded GFP^+ ^cells. **(A) **Scatter plot and linear regression analysis showing a lack of correlation between input membrane resistance (IR) and days post-injection (dpi) for neuroblasts migrating in the RMS. **(B) **A significant correlation was found in maturing GCs. Each point in the plot represents the values of one recorded GFP^+ ^cell. Click here for file

Additional file 4 **Figure S4**. Immunohistochemical staining for NKCC1 in control and shNKCC1 neuroblasts and GCs. **(A) **Upper panel: photomicrograph showing the immunolabeling for NKCC1 (red) and GFP^+ ^(green) on migrating neuroblasts in the RMS transduced with the control non-target sequence. Lower panel: product of the differences from the mean (PDM) analysis for the double staining, as described for Figure 2. (Ai) Upper panel: photomicrograph showing the immunolabeling for NKCC1 (red) on GFP^+ ^(green) neuroblasts located in the RMS and transduced with the shNKCC1 sequence. Lower panel: PDM analysis for the upper panel image. **(B) **Upper panel: photomicrograph showing the immunolabeling for NKCC1 (red) and GFP (green) on GCs transduced with the control non-target sequence. Lower panel: PDM analysis for the double labeling. (Bi) Upper panel: photomicrograph showing the immunolabeling for NKCC1 (red) in GFP^+ ^(green) neuroblasts located in the GCL and transduced with the shNKCC1 sequence. Lower panel: PDM analysis for the upper panel image. For (A,Ai,B,Bi) scale bars are 10 μm. Photographs in (A,Ai) were taken from cultures fixed at 6 dpi and in (B,Bi) at 7 dpi. **(C) **Normalized average (± standard error of the mean) number of neuroblasts and GCs showing correlated NKCC1 and GFP signals in cells transduced with the control and shNKCC1 sequences. Click here for file

Additional file 5 **Video 1**. Video showing the migration of neuroblasts transduced with the non-target control sequence in the RMS. The total duration of the session is 3 hours. Click here for file

Additional file 6 **Video 2**. Video showing the migration of neuroblasts transduced with the shNKCC1 sequence in the RMS. The total duration of the session is 3 hours. Click here for file

Additional file 7 **Figure S5**. Immunohistochemical staining for KCC2 in the organotypic slices 6 days after transduction with the shNKCC1- and GFP-encoding sequences. **(A) **Upper panel: photomicrograph of the RMS showing KCC2 immunostaining (red) in GFP^+ ^migrating neuroblasts (green). Lower panel: product of the differences from the mean (PDM) analysis for the immunostaining in (A), as explained in Figure 2. **(B) **Upper panel: photomicrograph showing KCC2 immunostaining (red) of GFP^+ ^maturing interneurons in the GCL. Lower panel: PDM analysis for the immunostaining in (B). Scale bars: 10 μm in (A) and 20 μm in (B). Click here for file

Additional file 8 **Figure S6**. The reversal potential of GABA_A_-mediated responses in neuroblasts and GCs did not evolve in the culture. **(A,B) **Scatter plot and linear regression analysis showing the lack of correlation between the reversal potential for GABA and days post-injection (dpi) in neuroblasts migrating in the RMS (A) and in GCs (B). Each point in the plot represents the values of one recorded GFP^+ ^cell. Click here for file

Additional file 9 **Figure S7**. Example traces showing the measurements of voltage-dependent K^+ ^current reversal in neuroblasts recorded in the cell attached configuration. Upper panel: three independent traces (gray) and their average (black) recorded in response to the voltage ramp depicted in the lower panel. **(A,B) **The blue dashed lines represent the linear fit applied to the average current trace and the black dashed lines indicate the corresponding voltage value for the reversal of K^+ ^voltage-dependent currents in a control cell (A) and in a shNKCC1 cell (B). Scale bars: 100 pA and 2 ms for (A) and 50 pA and 2 ms for (B). Click here for file

## References

[B1] Altman J Autoradiographic and histological studies of postnatal neurogenesis. IV. Cell proliferation and migration in the anterior forebrain, with special reference to persisting neurogenesis in the olfactory bulb J Comp Neurol 1969 137 433 457 10.1002/cne.901370404 5361244

[B2] Lledo PM Alonso M Grubb MS Adult neurogenesis and functional plasticity in neuronal circuits Nat Rev Neurosci 2006 7 179 193 10.1038/nrn1867 16495940

[B3] Lois C Alvarez-Buylla A Long-distance neuronal migration in the adult mammalian brain Science 1994 264 1145 1148 10.1126/science.8178174 8178174

[B4] Luskin MB Restricted proliferation and migration of postnatally generated neurons derived from the forebrain subventricular zone Neuron 1993 11 173 189 10.1016/0896-6273(93)90281-U 8338665

[B5] Hu H Rutishauser U A septum-derived chemorepulsive factor for migrating olfactory interneuron precursors Neuron 1996 16 933 940 10.1016/S0896-6273(00)80116-6 8630251

[B6] Jankovski A Sotelo C Subventricular zone-olfactory bulb migratory pathway in the adult mouse: cellular composition and specificity as determined by heterochronic and heterotopic transplantation J Comp Neurol 1996 371 376 396 10.1002/(SICI)1096-9861(19960729)371:3<376::AID-CNE3>3.0.CO;2-# 8842894

[B7] Murase S Horwitz AF Deleted in colorectal carcinoma and differentially expressed integrins mediate the directional migration of neural precursors in the rostral migratory stream J Neurosci 2002 22 3568 3579 11978833 10.1523/JNEUROSCI.22-09-03568.2002 PMC6758349

[B8] Hagg T Molecular regulation of adult CNS neurogenesis: an integrated view Trends Neurosci 2005 28 589 595 10.1016/j.tins.2005.08.009 16153715

[B9] Nguyen-Ba-Charvet KT Picard-Riera N Tessier-Lavigne M Baron-Van Evercooren A Sotelo C Chedotal A Multiple roles for slits in the control of cell migration in the rostral migratory stream J Neurosci 2004 24 1497 1506 10.1523/JNEUROSCI.4729-03.2004 14960623 PMC6730320

[B10] Bordey A Enigmatic GABAergic networks in adult neurogenic zones Brain Res Rev 2007 53 124 134 10.1016/j.brainresrev.2006.07.004 16949673

[B11] Ming GL Song H Adult neurogenesis in the mammalian central nervous system Annu Rev Neurosci 2005 28 223 250 10.1146/annurev.neuro.28.051804.101459 16022595

[B12] Bolteus AJ Bordey A GABA release and uptake regulate neuronal precursor migration in the postnatal subventricular zone J Neurosci 2004 24 7623 7631 10.1523/JNEUROSCI.1999-04.2004 15342728 PMC6729616

[B13] Wang DD Krueger DD Bordey A GABA depolarizes neuronal progenitors of the postnatal subventricular zone via GABAA receptor activation J Physiol 2003 550 785 800 10.1113/jphysiol.2003.042572 12807990 PMC2343064

[B14] Ben-Ari Y Cherubini E Corradetti R Gaiarsa JL Giant synaptic potentials in immature rat CA3 hippocampal neurones J Physiol 1989 416 303 325 2575165 10.1113/jphysiol.1989.sp017762 PMC1189216

[B15] Misgeld U Deisz RA Dodt HU Lux HD The role of chloride transport in postsynaptic inhibition of hippocampal neurons Science 1986 232 1413 1415 10.1126/science.2424084 2424084

[B16] Mueller AL Taube JS Schwartzkroin PA Development of hyperpolarizing inhibitory postsynaptic potentials and hyperpolarizing response to gamma-aminobutyric acid in rabbit hippocampus studied in vitro J Neurosci 1984 4 860 867 6707735 10.1523/JNEUROSCI.04-03-00860.1984 PMC6564832

[B17] Rivera C Voipio J Payne JA Ruusuvuori E Lahtinen H Lamsa K Pirvola U Saarma M Kaila K The K+/Cl- co-transporter KCC2 renders GABA hyperpolarizing during neuronal maturation Nature 1999 397 251 255 10.1038/16697 9930699

[B18] Owens DF Boyce LH Davis MB Kriegstein AR Excitatory GABA responses in embryonic and neonatal cortical slices demonstrated by gramicidin perforated-patch recordings and calcium imaging J Neurosci 1996 16 6414 6423 8815920 10.1523/JNEUROSCI.16-20-06414.1996 PMC6578913

[B19] Yamada J Okabe A Toyoda H Kilb W Luhmann HJ Fukuda A Cl- uptake promoting depolarizing GABA actions in immature rat neocortical neurones is mediated by NKCC1 J Physiol 2004 557 829 841 10.1113/jphysiol.2004.062471 15090604 PMC1665166

[B20] Delpy A Allain AE Meyrand P Branchereau P NKCC1 cotransporter inactivation underlies embryonic development of chloride-mediated inhibition in mouse spinal motoneuron J Physiol 2008 586 1059 1075 10.1113/jphysiol.2007.146993 18096599 PMC2375629

[B21] Ge S Goh EL Sailor KA Kitabatake Y Ming GL Song H GABA regulates synaptic integration of newly generated neurons in the adult brain Nature 2006 439 589 593 10.1038/nature04404 16341203 PMC1420640

[B22] Plotkin MD Snyder EY Hebert SC Delpire E Expression of the Na-K-2Cl cotransporter is developmentally regulated in postnatal rat brains: a possible mechanism underlying GABA's excitatory role in immature brain J Neurobiol 1997 33 781 795 10.1002/(SICI)1097-4695(19971120)33:6<781::AID-NEU6>3.0.CO;2-5 9369151

[B23] Wang DD Kriegstein AR GABA regulates excitatory synapse formation in the neocortex via NMDA receptor activation J Neurosci 2008 28 5547 5558 10.1523/JNEUROSCI.5599-07.2008 18495889 PMC2684685

[B24] Ben-Ari Y Excitatory actions of gaba during development: the nature of the nurture Nat Rev Neurosci 2002 3 728 739 10.1038/nrn920 12209121

[B25] Gascon E Dayer AG Sauvain MO Potter G Jenny B De Roo M Zgraggen E Demaurex N Muller D Kiss JZ GABA regulates dendritic growth by stabilizing lamellipodia in newly generated interneurons of the olfactory bulb J Neurosci 2006 26 12956 12966 10.1523/JNEUROSCI.4508-06.2006 17167085 PMC6674946

[B26] Mejia-Gervacio S Marty A Control of interneurone firing pattern by axonal autoreceptors in the juvenile rat cerebellum J Physiol 2006 571 43 55 10.1113/jphysiol.2005.101675 16339174 PMC1805651

[B27] Obrietan K van den Pol AN Growth cone calcium elevation by GABA J Comp Neurol 1996 372 167 175 10.1002/(SICI)1096-9861(19960819)372:2<167::AID-CNE1>3.0.CO;2-1 8863123

[B28] Alonso M Ortega-Perez I Grubb MS Bourgeois JP Charneau P Lledo PM Turning astrocytes from the rostral migratory stream into neurons: a role for the olfactory sensory organ J Neurosci 2008 28 11089 11102 10.1523/JNEUROSCI.3713-08.2008 18945916 PMC6671355

[B29] Imayoshi I Sakamoto M Ohtsuka T Takao K Miyakawa T Yamaguchi M Mori K Ikeda T Itohara S Kageyama R Roles of continuous neurogenesis in the structural and functional integrity of the adult forebrain Nat Neurosci 2008 11 1153 1161 10.1038/nn.2185 18758458

[B30] Kishi K Golgi studies on the development of granule cells of the rat olfactory bulb with reference to migration in the subependymal layer J Comp Neurol 1987 258 112 124 10.1002/cne.902580109 3571532

[B31] Carleton A Petreanu LT Lansford R Alvarez-Buylla A Lledo PM Becoming a new neuron in the adult olfactory bulb Nat Neurosci 2003 6 507 518 12704391 10.1038/nn1048

[B32] Payne JA Rivera C Voipio J Kaila K Cation-chloride co-transporters in neuronal communication, development and trauma Trends Neurosci 2003 26 199 206 10.1016/S0166-2236(03)00068-7 12689771

[B33] Russell JM Sodium-potassium-chloride cotransport Physiol Rev 2000 80 211 276 10617769 10.1152/physrev.2000.80.1.211

[B34] Jagasia R Steib K Englberger E Herold S Faus-Kessler T Saxe M Gage FH Song H Lie DC GABA-cAMP response element-binding protein signaling regulates maturation and survival of newly generated neurons in the adult hippocampus J Neurosci 2009 29 7966 7977 10.1523/JNEUROSCI.1054-09.2009 19553437 PMC2776747

[B35] Ceccarelli B Hurlbut WP Ca2+-dependent recycling of synaptic vesicles at the frog neuromuscular junction J Cell Biol 1980 87 297 303 10.1083/jcb.87.1.297 6252215 PMC2110728

[B36] Tzeng MC Siekevitz P The effect of the purified major protein factor (alpha-latrotoxin) of black widow spider venom on the release of acetylcholine and norepinephrine from mouse cerebral cortex slices Brain Res 1978 139 190 196 10.1016/0006-8993(78)90073-2 620349

[B37] Brickley SG Cull-Candy SG Farrant M Development of a tonic form of synaptic inhibition in rat cerebellar granule cells resulting from persistent activation of GABAA receptors J Physiol 1996 497 753 759 9003560 10.1113/jphysiol.1996.sp021806 PMC1160971

[B38] Verheugen JA Fricker D Miles R Noninvasive measurements of the membrane potential and GABAergic action in hippocampal interneurons J Neurosci 1999 19 2546 2555 10087068 10.1523/JNEUROSCI.19-07-02546.1999 PMC6786065

[B39] Brumback AC Staley KJ Thermodynamic regulation of NKCC1-mediated Cl- cotransport underlies plasticity of GABA(A) signaling in neonatal neurons J Neurosci 2008 28 1301 1312 10.1523/JNEUROSCI.3378-07.2008 18256250 PMC6671583

[B40] Cancedda L Fiumelli H Chen K Poo MM Excitatory GABA action is essential for morphological maturation of cortical neurons *in vivo* J Neurosci 2007 27 5224 5235 10.1523/JNEUROSCI.5169-06.2007 17494709 PMC6672363

[B41] Sipila ST Huttu K Yamada J Afzalov R Voipio J Blaesse P Kaila K Compensatory enhancement of intrinsic spiking upon NKCC1 disruption in neonatal hippocampus J Neurosci 2009 29 6982 6988 10.1523/JNEUROSCI.0443-09.2009 19474325 PMC6665606

[B42] Rakowski RF Gadsby DC De Weer P Stoichiometry and voltage dependence of the sodium pump in voltage-clamped, internally dialyzed squid giant axon J Gen Physiol 1989 93 903 941 10.1085/jgp.93.5.903 2544655 PMC2216238

[B43] Liu X Wang Q Haydar TF Bordey A Nonsynaptic GABA signaling in postnatal subventricular zone controls proliferation of GFAP-expressing progenitors Nat Neurosci 2005 8 1179 1187 10.1038/nn1522 16116450 PMC1380263

[B44] Platel JC Heintz T Young S Gordon V Bordey A Tonic activation of GLUK5 kainate receptors decreases neuroblast migration in whole-mounts of the subventricular zone J Physiol 2008 586 3783 3793 10.1113/jphysiol.2008.155879 18565997 PMC2538932

[B45] Heck N Kilb W Reiprich P Kubota H Furukawa T Fukuda A Luhmann HJ GABA-A receptors regulate neocortical neuronal migration in vitro and *in vivo* Cereb Cortex 2007 17 138 148 10.1093/cercor/bhj135 16452638

[B46] Petreanu L Alvarez-Buylla A Maturation and death of adult-born olfactory bulb granule neurons: role of olfaction J Neurosci 2002 22 6106 6113 12122071 10.1523/JNEUROSCI.22-14-06106.2002 PMC6757952

[B47] Frazier-Cierpial L Brunjes PC Early postnatal cellular proliferation and survival in the olfactory bulb and rostral migratory stream of normal and unilaterally odor-deprived rats J Comp Neurol 1989 289 481 492 10.1002/cne.902890312 2808782

[B48] Jankovski A Garcia C Soriano E Sotelo C Proliferation, migration and differentiation of neuronal progenitor cells in the adult mouse subventricular zone surgically separated from its olfactory bulb Eur J Neurosci 1998 10 3853 3868 10.1046/j.1460-9568.1998.00397.x 9875362

[B49] Parent JM Valentin VV Lowenstein DH Prolonged seizures increase proliferating neuroblasts in the adult rat subventricular zone-olfactory bulb pathway J Neurosci 2002 22 3174 3188 11943819 10.1523/JNEUROSCI.22-08-03174.2002 PMC6757526

[B50] Arvidsson A Collin T Kirik D Kokaia Z Lindvall O Neuronal replacement from endogenous precursors in the adult brain after stroke Nat Med 2002 8 963 970 10.1038/nm747 12161747

[B51] Balena T Woodin MA Coincident pre- and postsynaptic activity downregulates NKCC1 to hyperpolarize E(Cl) during development Eur J Neurosci 2008 27 2402 2412 10.1111/j.1460-9568.2008.06194.x 18430034

[B52] Fiumelli H Cancedda L Poo MM Modulation of GABAergic transmission by activity via postsynaptic Ca2+-dependent regulation of KCC2 function Neuron 2005 48 773 786 10.1016/j.neuron.2005.10.025 16337915

[B53] Tanaka Y Tozuka Y Takata T Shimazu N Matsumura N Ohta A Hisatsune T Excitatory GABAergic activation of cortical dividing glial cells Cereb Cortex 2009 19 2181 2195 10.1093/cercor/bhn238 19131437

[B54] Akiba Y Cave JW Akiba N Langley B Ratan RR Baker H Histone deacetylase inhibitors de-repress tyrosine hydroxylase expression in the olfactory bulb and rostral migratory stream Biochem Biophys Res Commun 2010 393 673 677 10.1016/j.bbrc.2010.02.054 20170631 PMC2848448

[B55] Schoppa NE A novel local circuit in the olfactory bulb involving an old short-axon cell Neuron 2006 49 783 784 10.1016/j.neuron.2006.03.005 16543124

[B56] Wang C Ohno K Furukawa T Ueki T Ikeda M Fukuda A Sato K Differential expression of KCC2 accounts for the differential GABA responses between relay and intrinsic neurons in the early postnatal rat olfactory bulb Eur J Neurosci 2005 21 1449 1455 10.1111/j.1460-9568.2005.03975.x 15813956

[B57] Chavas J Marty A Coexistence of excitatory and inhibitory GABA synapses in the cerebellar interneuron network J Neurosci 2003 23 2019 2031 12657660 10.1523/JNEUROSCI.23-06-02019.2003 PMC6742031

[B58] Banke TG McBain CJ GABAergic input onto CA3 hippocampal interneurons remains shunting throughout development J Neurosci 2006 26 11720 11725 10.1523/JNEUROSCI.2887-06.2006 17093093 PMC6674795

[B59] Obata K Transmitter sensitivities of some nerve and muscle cells in culture Brain Res 1974 73 71 88 10.1016/0006-8993(74)91008-7 4366425

[B60] Blaesse P Airaksinen MS Rivera C Kaila K Cation-chloride cotransporters and neuronal function Neuron 2009 61 820 838 10.1016/j.neuron.2009.03.003 19323993

[B61] Li H Khirug S Cai C Ludwig A Blaesse P Kolikova J Afzalov R Coleman SK Lauri S Airaksinen MS Keinänen K Khiroug L Saarma M Kaila K Rivera C KCC2 interacts with the dendritic cytoskeleton to promote spine development Neuron 2007 56 1019 1033 10.1016/j.neuron.2007.10.039 18093524

[B62] Belluzzi O Benedusi M Ackman J LoTurco JJ Electrophysiological differentiation of new neurons in the olfactory bulb J Neurosci 2003 23 10411 10418 14614100 10.1523/JNEUROSCI.23-32-10411.2003 PMC6741027

[B63] Stewart RR Hoge GJ Zigova T Luskin MB Neural progenitor cells of the neonatal rat anterior subventricular zone express functional GABA(A) receptors J Neurobiol 2002 50 305 322 10.1002/neu.10038 11891665

[B64] Stoppini L Buchs PA Muller D A simple method for organotypic cultures of nervous tissue J Neurosci Methods 1991 37 173 182 10.1016/0165-0270(91)90128-M 1715499

[B65] Avale ME Faure P Pons S Robledo P Deltheil T David DJ Gardier AM Maldonado R Granon S Changeux JP Maskos U Interplay of beta2* nicotinic receptors and dopamine pathways in the control of spontaneous locomotion Proc Natl Acad Sci USA 2008 105 15991 15996 10.1073/pnas.0807635105 18832468 PMC2559801

[B66] Kyrozis A Reichling DB Perforated-patch recording with gramicidin avoids artifactual changes in intracellular chloride concentration J Neurosci Methods 1995 57 27 35 10.1016/0165-0270(94)00116-X 7540702

[B67] Ehrlich I Lohrke S Friauf E Shift from depolarizing to hyperpolarizing glycine action in rat auditory neurones is due to age-dependent Cl- regulation J Physiol 1999 520 121 137 10.1111/j.1469-7793.1999.00121.x 10517806 PMC2269580

[B68] Khawaled R Bruening-Wright A Adelman JP Maylie J Bicuculline block of small-conductance calcium-activated potassium channels Pflugers Arch 1999 438 314 321 10.1007/s004240050915 10398861

[B69] Li Q Lau A Morris TJ Guo L Fordyce CB Stanley EF A syntaxin 1, Galpha(o), and N-type calcium channel complex at a presynaptic nerve terminal: analysis by quantitative immunocolocalization J Neurosci 2004 24 4070 4081 10.1523/JNEUROSCI.0346-04.2004 15102922 PMC6729428

[B70] The R Project for Statistical Computing http://www.r-project.org/

