# Supplementary figures and images for: NKCC1 controls GABAergic signaling and neuroblast migration in the postnatal forebrain
Source: Neural Dev. 2011 Feb 1;6:4. doi: 10.1186/1749-8104-6-4 (PMC3038882; doi:10.1186/1749-8104-6-4)

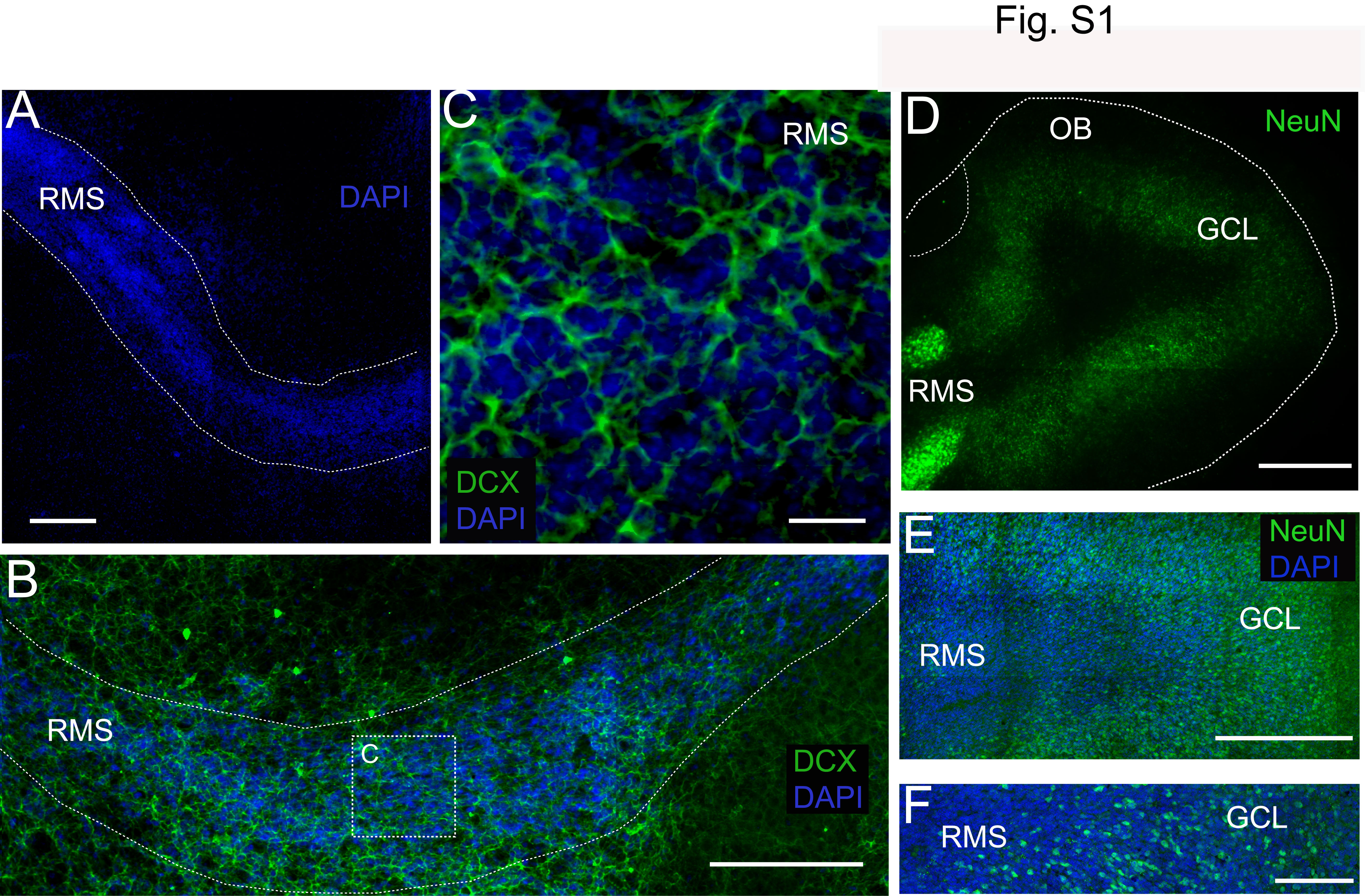

Supplement: Additional file 1 — Figure S1. Immunohistochemical staining for doublecortin (DCX) and neuronal nuclei marker (NeuN) in 6-div organotypic cultures. (A) Low magnification image (10×) from a sagittal organotypic slice stained with DAPI (blue) depicting the cellular nuclei in the rostral migratory stream (RMS), as delimited by dashed lines. (B) DCX (green) and DAPI labeling in the RMS elbow area. Inset: approximate location of the area shown in (C). (C) High magnification detail of the RMS double stained for DCX and DAPI. (D) Low magnification image showing the distribution of NeuN-expressing cells (green) in the granule cell layer (GCL) and absence of positive cells in the RMS in a sagittal organotypic slice of the OB. Outlined by dashed lines, the borders of the OB and the accessory OB are depicted for reference. (E) High magnification detail of the GCL region and OB RMS showing NeuN-expressing cells in the former and the cellular nuclei stained with DAPI. (F) Higher magnification detail of the OB RMS and GCL double stained for DAPI and NeuN. Scale bars: 250 μm for (A,E), 200 μm for (B,F), 20 μm for (C), and 500 μm for (D). [file 1749-8104-6-4-S1.JPEG]

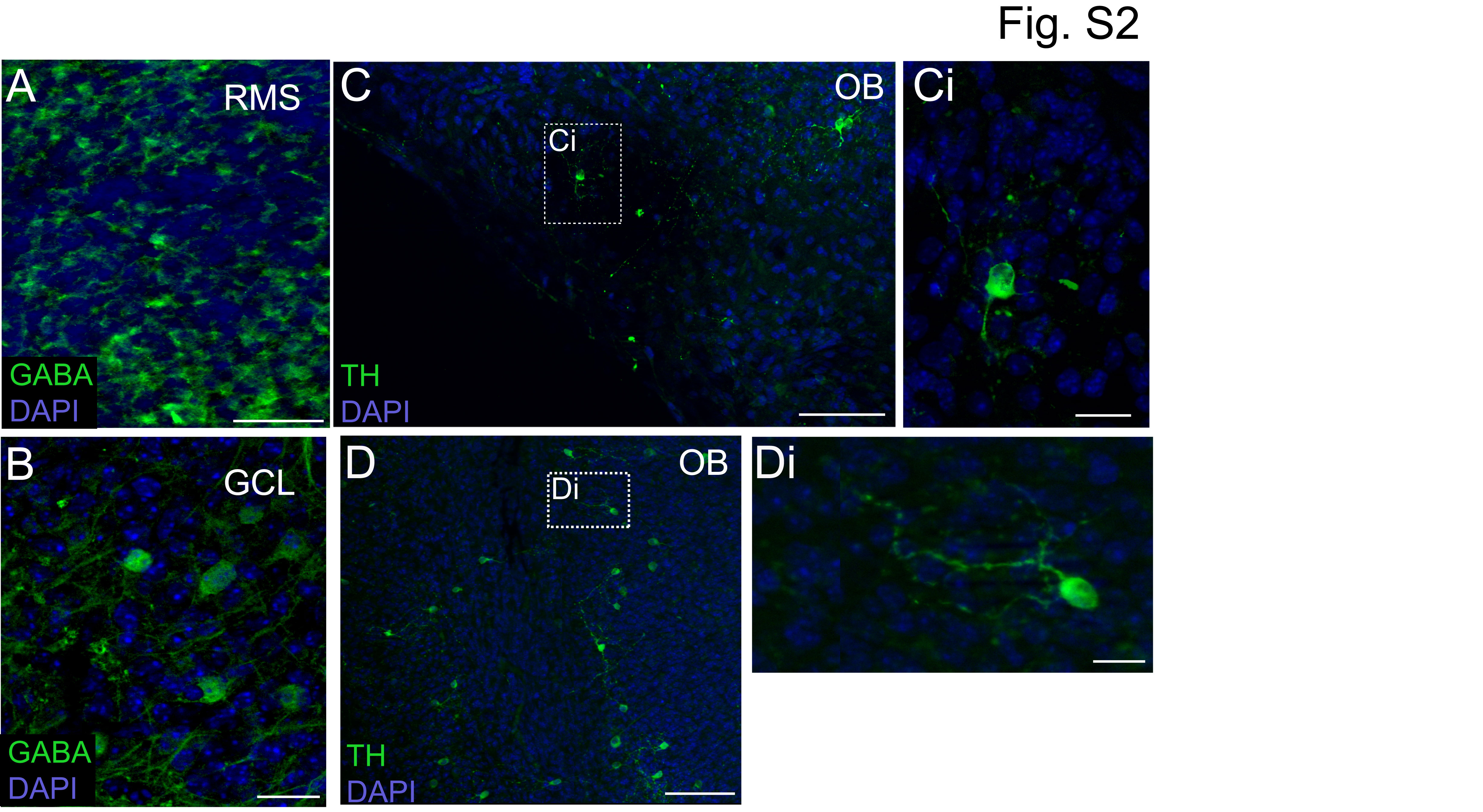

Supplement: Additional file 2 — Figure S2. Immunohistochemical staining for GABA and tyrosine hydroxylase (TH) in 6-div organotypic cultures. (A,B) Photomicrograph showing GABAergic cells (green) and cellular nuclei co-stained with DAPI (blue) in the RMS (A), and in the GCL regions (B). (C) Photomicrograph showing the presence of TH-positive cells (green) surrounding a glomerulus in the OB, in an organotypic slice co-stained with DAPI. Inset: approximate location of the region magnified in (Ci). (Ci) High magnification photomicrograph showing a TH-positive cell costained with DAPI in an OB glomerulus. (D) Photomicrograph showing TH-expressing cells in the OB of an organotypic slice. Inset: approximate location of the region depicted in (Di). (Di) High magnification photomicrograph of a TH-expressing cell double labeled with DAPI in the OB. Scale bars: 50 μm for (A), 20 μm for (B), 100 μm in (C,D), 20 μm in (Ci,Di). [file 1749-8104-6-4-S2.JPEG]

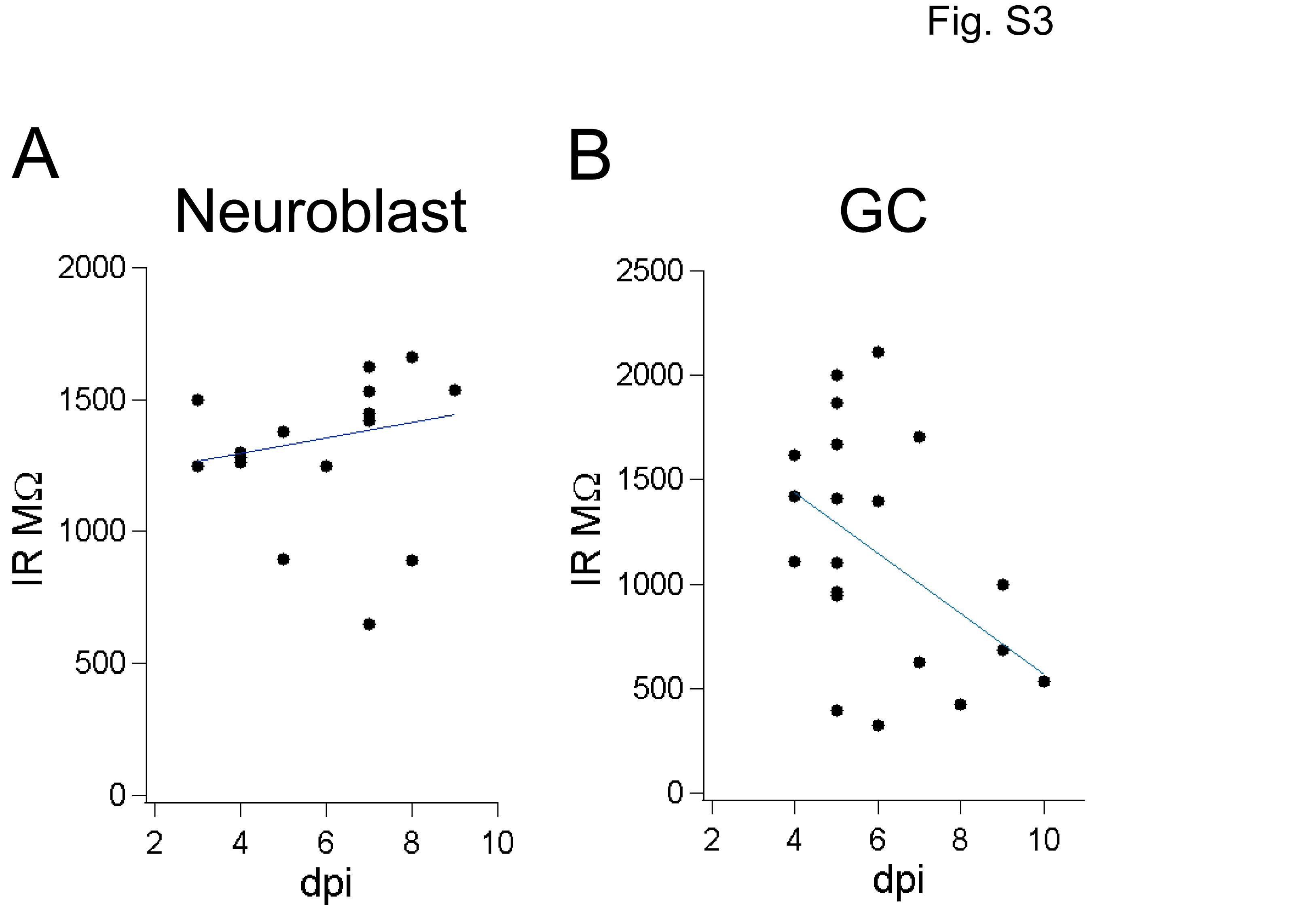

Supplement: Additional file 3 — Figure S3. Functional maturation of recorded GFP+ cells. (A) Scatter plot and linear regression analysis showing a lack of correlation between input membrane resistance (IR) and days post-injection (dpi) for neuroblasts migrating in the RMS. (B) A significant correlation was found in maturing GCs. Each point in the plot represents the values of one recorded GFP+ cell. [file 1749-8104-6-4-S3.JPEG]

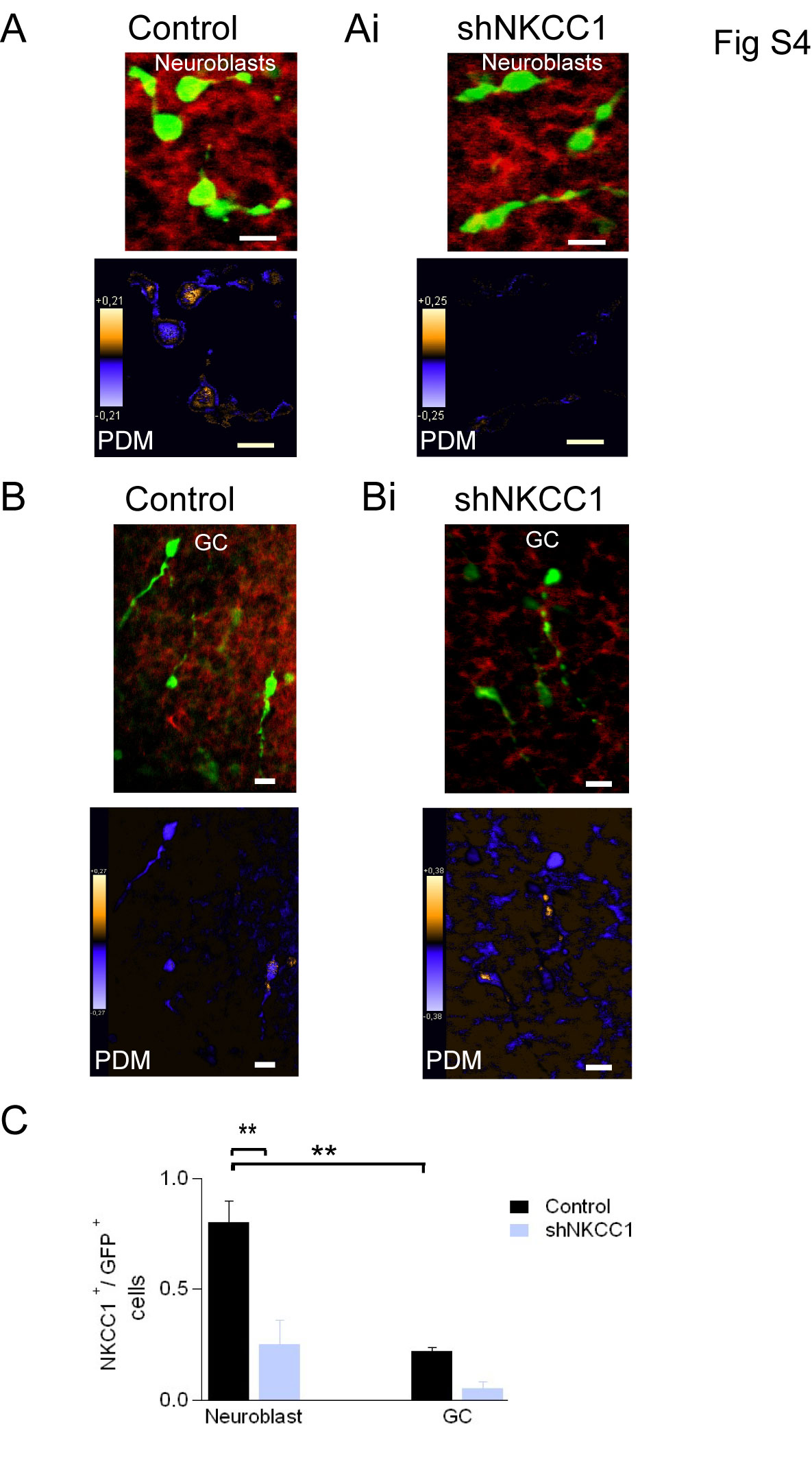

Supplement: Additional file 4 — Figure S4. Immunohistochemical staining for NKCC1 in control and shNKCC1 neuroblasts and GCs. (A) Upper panel: photomicrograph showing the immunolabeling for NKCC1 (red) and GFP+ (green) on migrating neuroblasts in the RMS transduced with the control non-target sequence. Lower panel: product of the differences from the mean (PDM) analysis for the double staining, as described for Figure 2. (Ai) Upper panel: photomicrograph showing the immunolabeling for NKCC1 (red) on GFP+ (green) neuroblasts located in the RMS and transduced with the shNKCC1 sequence. Lower panel: PDM analysis for the upper panel image. (B) Upper panel: photomicrograph showing the immunolabeling for NKCC1 (red) and GFP (green) on GCs transduced with the control non-target sequence. Lower panel: PDM analysis for the double labeling. (Bi) Upper panel: photomicrograph showing the immunolabeling for NKCC1 (red) in GFP+ (green) neuroblasts located in the GCL and transduced with the shNKCC1 sequence. Lower panel: PDM analysis for the upper panel image. For (A,Ai,B,Bi) scale bars are 10 μm. Photographs in (A,Ai) were taken from cultures fixed at 6 dpi and in (B,Bi) at 7 dpi. (C) Normalized average (± standard error of the mean) number of neuroblasts and GCs showing correlated NKCC1 and GFP signals in cells transduced with the control and shNKCC1 sequences. [file 1749-8104-6-4-S4.JPEG]

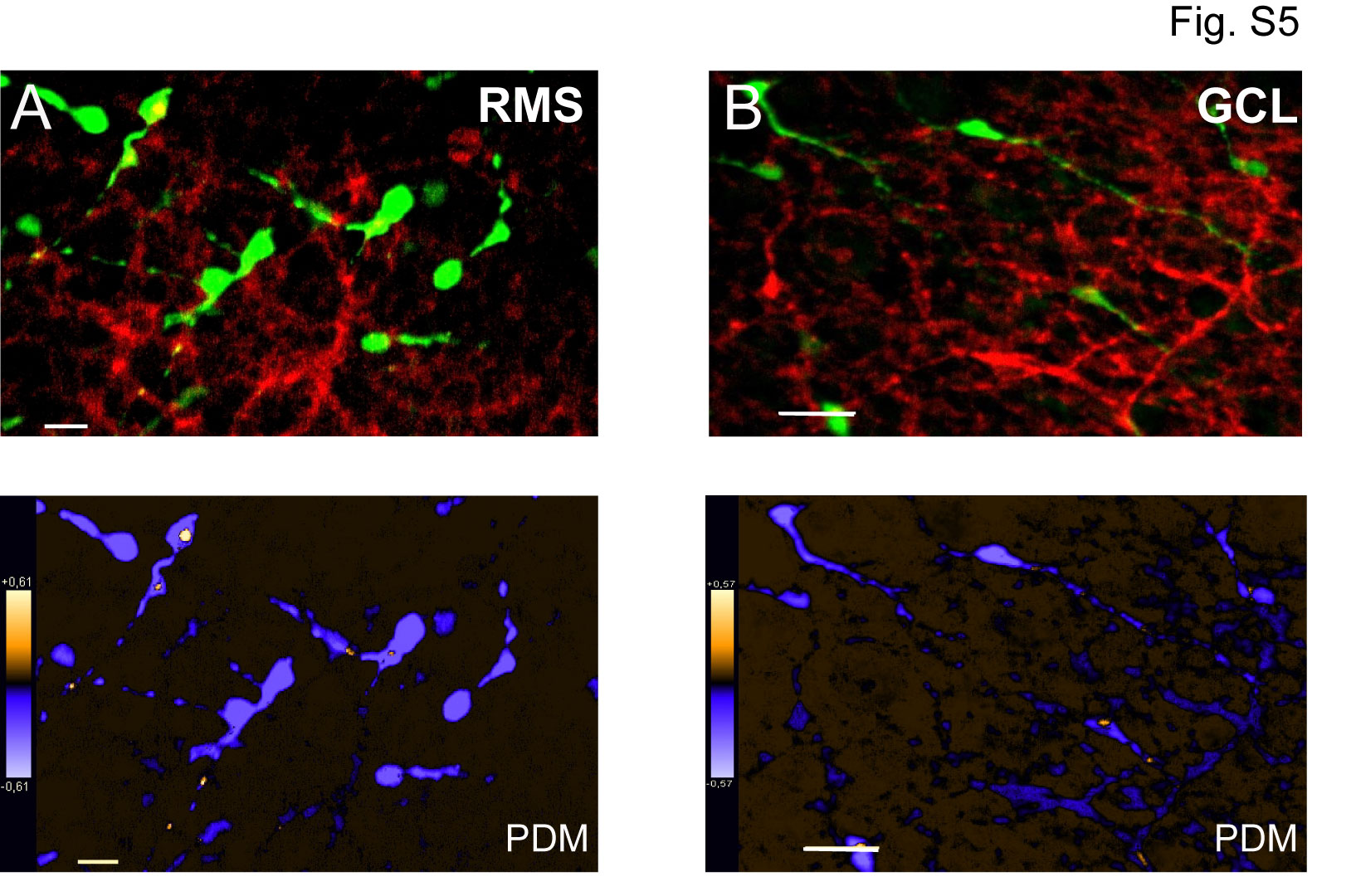

Supplement: Additional file 7 — Figure S5. Immunohistochemical staining for KCC2 in the organotypic slices 6 days after transduction with the shNKCC1- and GFP-encoding sequences. (A) Upper panel: photomicrograph of the RMS showing KCC2 immunostaining (red) in GFP+ migrating neuroblasts (green). Lower panel: product of the differences from the mean (PDM) analysis for the immunostaining in (A), as explained in Figure 2. (B) Upper panel: photomicrograph showing KCC2 immunostaining (red) of GFP+ maturing interneurons in the GCL. Lower panel: PDM analysis for the immunostaining in (B). Scale bars: 10 μm in (A) and 20 μm in (B). [file 1749-8104-6-4-S7.JPEG]

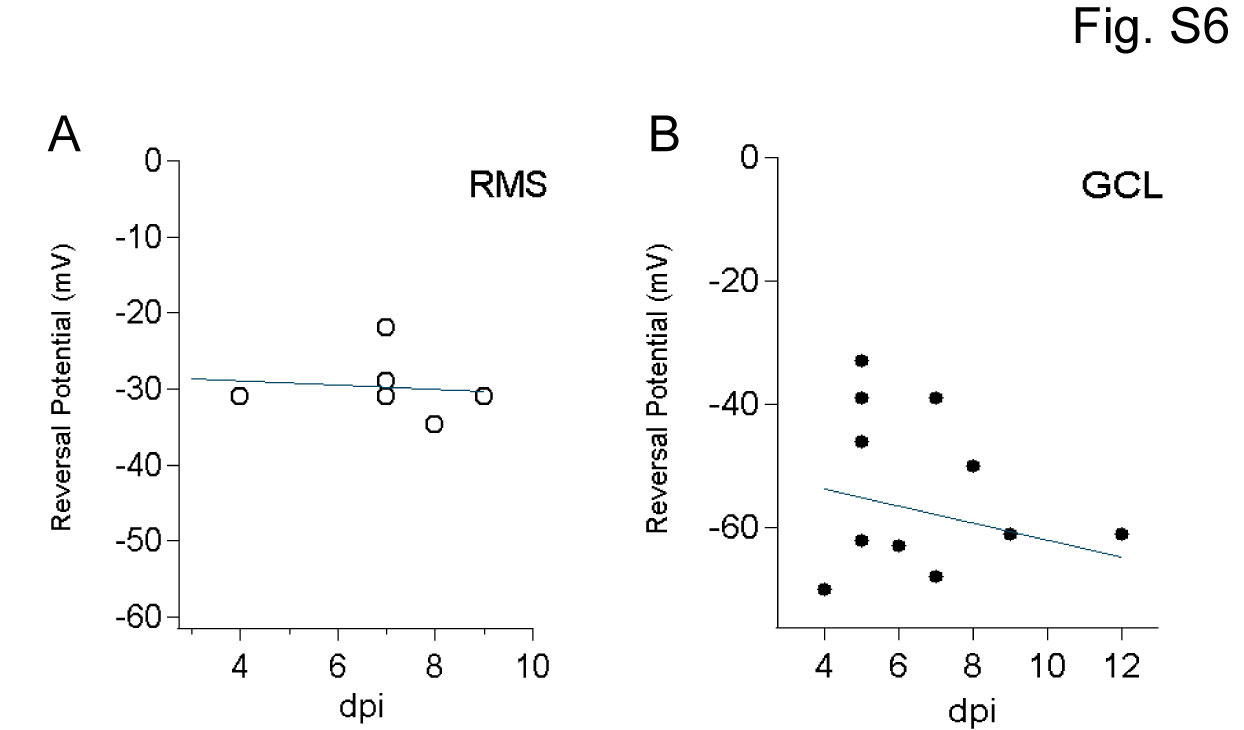

Supplement: Additional file 8 — Figure S6. The reversal potential of GABAA-mediated responses in neuroblasts and GCs did not evolve in the culture. (A,B) Scatter plot and linear regression analysis showing the lack of correlation between the reversal potential for GABA and days post-injection (dpi) in neuroblasts migrating in the RMS (A) and in GCs (B). Each point in the plot represents the values of one recorded GFP+ cell. [file 1749-8104-6-4-S8.JPEG]

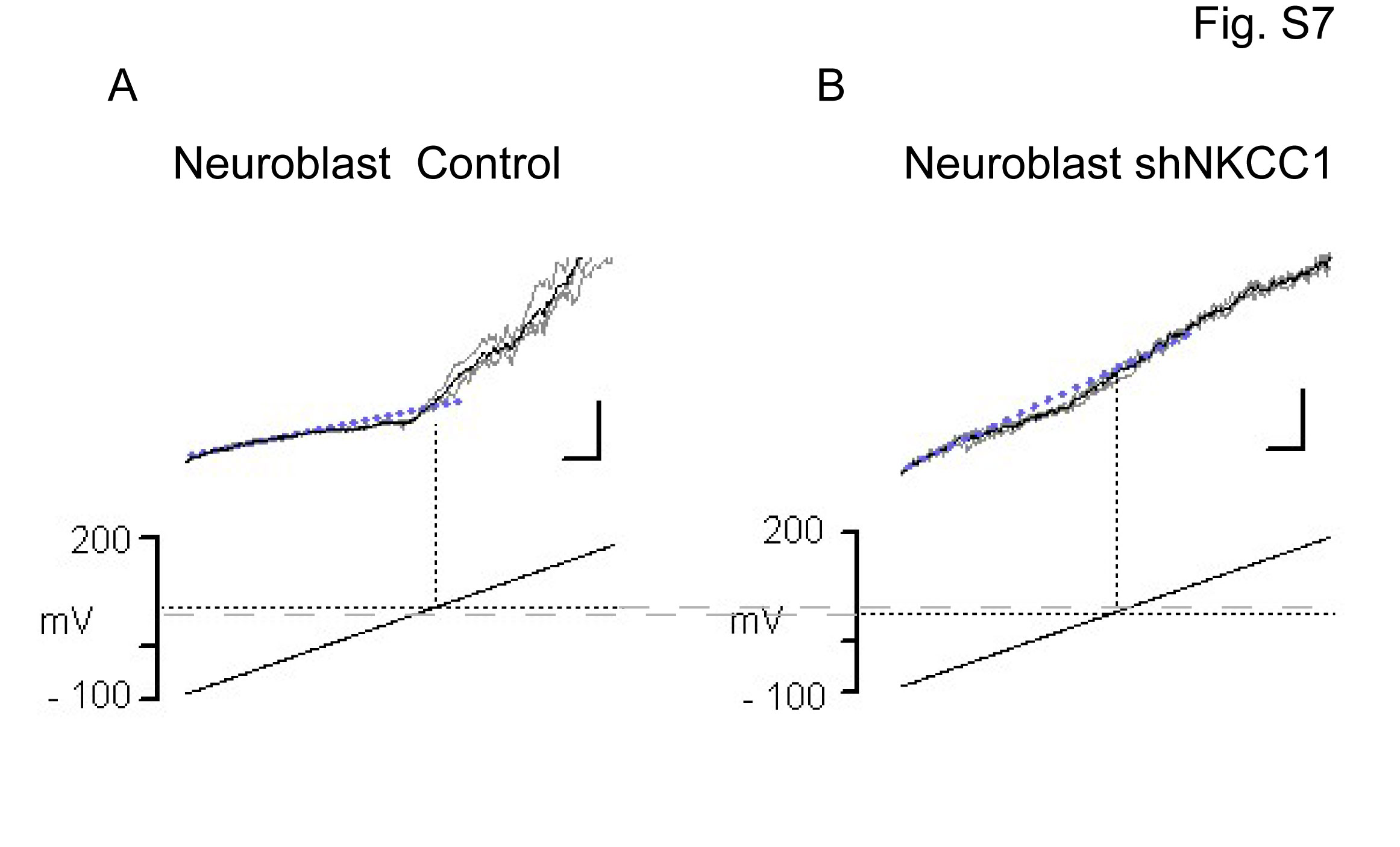

Supplement: Additional file 9 — Figure S7. Example traces showing the measurements of voltage-dependent K+ current reversal in neuroblasts recorded in the cell attached configuration. Upper panel: three independent traces (gray) and their average (black) recorded in response to the voltage ramp depicted in the lower panel. (A,B) The blue dashed lines represent the linear fit applied to the average current trace and the black dashed lines indicate the corresponding voltage value for the reversal of K+ voltage-dependent currents in a control cell (A) and in a shNKCC1 cell (B). Scale bars: 100 pA and 2 ms for (A) and 50 pA and 2 ms for (B). [file 1749-8104-6-4-S9.JPEG]
